# Challenges in multi-task learning for fMRI-based diagnosis: Benefits for psychiatric conditions and CNVs would likely require thousands of patients

**DOI:** 10.1162/imag_a_00222

**Published:** 2024-07-26

**Authors:** Annabelle Harvey, Clara A. Moreau, Kuldeep Kumar, Guillaume Huguet, Sebastian G.W. Urchs, Hanad Sharmarke, Khadije Jizi, Charles-Olivier Martin, Nadine Younis, Petra Tamer, Jean-Louis Martineau, Pierre Orban, Ana Isabel Silva, Jeremy Hall, Marianne B.M. van den Bree, Michael J. Owen, David E.J. Linden, Sarah Lippé, Carrie E. Bearden, Guillaume Dumas, Sébastien Jacquemont, Pierre Bellec

**Affiliations:** Department of Computer Science and Operational Research, University of Montréal, Montréal, Canada; Centre de recherche de l’institut universitaire de gériatrie de Montréal, Montréal, Canada; Centre de recherche du CHU Sainte-Justine, Montréal, Canada; Mark and Mary Stevens Neuroimaging and Informatics Institute, University of Southern California, Los Angeles, CA, United States; Keck School of Medicine, University of Southern California, Marina del Rey, CA, United States; Laboratory for Brain Simulation and Exploration, Université de Montréal, Montréal, Canada; Centre de recherche de l'Institut universitaire en santé mentale de Montréal, Montréal, Canada; Department of Psychiatry and Addictology, Université de Montréal, Montréal, Canada; Center for Magnetic Resonance Research, Department of Radiology, University of Minnesota, Minneapolis, MN, United States; Neuroscience and Mental Health Innovation Institute, Cardiff University, Cardiff, United Kingdom; Division of Psychological Medicine and Clinical Neurosciences, School of Medicine, Cardiff University, Cardiff, United Kingdom; Centre for Neuropsychiatric Genetics and Genomics, Cardiff University, Cardiff, United Kingdom; Institute for Mental Health and Neuroscience, Faculty of Health, Medicine and Life Sciences, Maastricht University, Maastricht, Netherlands; Department of Psychology, Université de Montréal, Montréal, Canada; Department of Psychiatry and Biobehavioral Sciences, Semel Institute for Neuroscience and Human Behavior, University of California, Los Angeles, CA, United States; Department of Psychology, University of California, Los Angeles, CA, United States; Department of Psychiatry, Université de Montréal, Montréal, Canada; Mila – Québec AI Institute, Université de Montréal, Montréal, Canada; Department of Pediatrics, Faculty of Medicine, Université de Montréal, Montréal, Canada

**Keywords:** machine learning, multi-task learning, multi-site data, fMRI, CNVs, psychiatric conditions

## Abstract

There is a growing interest in using machine learning (ML) models to perform automatic diagnosis of psychiatric conditions; however, generalising the prediction of ML models to completely independent data can lead to sharp decrease in performance. Patients with different psychiatric diagnoses have traditionally been studied independently, yet there is a growing recognition of neuroimaging signatures shared across them as well as rare genetic copy number variants (CNVs). In this work, we assess the potential of multi-task learning (MTL) to improve accuracy by characterising multiple related conditions with a single model, making use of information shared across diagnostic categories and exposing the model to a larger and more diverse dataset. As a proof of concept, we first established the efficacy of MTL in a context where there is clearly information shared across tasks: the same target (age or sex) is predicted at different sites of data collection in a large functional magnetic resonance imaging (fMRI) dataset compiled from multiple studies. MTL generally led to substantial gains relative to independent prediction at each site. Performing scaling experiments on the UK Biobank, we observed that performance was highly dependent on sample size: for large sample sizes (N > 6000) sex prediction was better using MTL across three sites (N = K per site) than prediction at a single site (N = 3K), but for small samples (N < 500) MTL was actually detrimental for age prediction. We then used established machine-learning methods to benchmark the diagnostic accuracy of each of the 7 CNVs (N = 19–103) and 4 psychiatric conditions (N = 44–472) independently, replicating the accuracy previously reported in the literature on psychiatric conditions. We observed that MTL hurt performance when applied across the full set of diagnoses, and complementary analyses failed to identify pairs of conditions which would benefit from MTL. Taken together, our results show that if a successful multi-task diagnostic model of psychiatric conditions were to be developed with resting-state fMRI, it would likely require datasets with thousands of patients across different diagnoses.

## 1 Introduction

There is a growing interest in using machine learning (ML) models to perform automatic diagnosis of psychiatric conditions. Unlike group-level mass-univariate analyses, ML models identify multivariate patterns that characterise a condition by learning to distinguish patients from control subjects at the individual level. While many studies have reported promising results (Iyortsuun et al., 2023), generalising the prediction of ML models to completely independent data can lead to a sharp decrease in performance, as was recently evidenced for clinical trial stratification (Chekroud et al., 2024). This is due in large part to the massive biological heterogeneity that exists within the current diagnostic categories, which are based on behavioural symptoms alone (Pacheco et al., 2022). High rates of comorbidities (Katzman et al., 2017;McElroy, 2004;Simonoff et al., 2008;Tsai & Rosenheck, 2013) among psychiatric disorders, as well as genetic and symptoms overlap (Romero et al., 2022), and shared neuroimaging signatures (Vanes & Dolan, 2021;Xie et al., 2023) support the existence of latent factors that ignore diagnostic boundaries. Multi-task learning (MTL) is an ML framework that has the potential to improve prediction by characterising multiple related conditions with a single model, making use of information shared across diagnostic categories and exposing the model to a larger and more diverse dataset. Useful transdiagnostic information for MTL may also be found in rare genetic mutations, called copy number variants (CNVs), some of which confer a high risk for a range of psychiatric conditions (Marshall et al., 2017;Rees & Kirov, 2021;Sanders et al., 2019;Satterstrom et al., 2020). CNVs have a large impact on brain structure and function (Modenato et al., 2021;Moreau, Ching, et al., 2021;Moreau, Raznahan, et al., 2021;Moreau et al., 2020;Sønderby et al., 2022), which converges with neuroimaging brain signatures associated with psychiatric disorders (Moreau, Raznahan, et al., 2021). In this work, we aimed to assess the potential of MTL to exploit the relationships between a range of psychiatric and CNV conditions to improve the performance of automatic diagnosis. To this end, we compiled a resting state functional magnetic resonance imaging (rs-fMRI) dataset of 7 CNVs, which have never previously been studied in the ML context, and 4 psychiatric disorders.

ML models aim to identify patterns and mechanisms across regions in the brain that characterise a condition and provide scores for diagnosis at the individual level (Linn et al., 2016). While genetic variants conferring risk for psychiatric conditions have yet to be studied in the ML context, many studies have applied ML methods to automatically diagnose psychiatric conditions using rs-fMRI biomarkers, including schizophrenia (SZ) (Bassett et al., 2012;Kim et al., 2016;Venkataraman et al., 2012), autism spectrum disorder (ASD) (Abraham et al., 2017;Heinsfeld et al., 2018;Khosla et al., 2018;Nielsen et al., 2013;Traut et al., 2022), attention-deficit/hyperactivity disorder (ADHD) (Eloyan et al., 2012;J. Li et al., 2020;Z. Wang et al., 2023), and bipolar disorder (BIP) (Rashid et al., 2016;H. Wang et al., 2022). However, accuracy of automatic diagnosis reported in the literature should be interpreted with caution, as the majority of studies to date have been performed on data collected from a single site with a small sample size (Orban et al., 2018). Sample size is a crucial factor in training ML models. Accuracy of prediction generally increases with the number of subjects in studies that examine the impact of sample size (Schulz et al., 2020;Traut et al., 2022). While more subjects allow the model to learn a better signature for a given condition, using small samples can yield deceptively high prediction accuracy due to overfitting (where a model memorises aspects of the dataset used to train it, but fails to generalise to new data). In a meta-analysis (including studies on ASD and SZ among other psychiatric diagnoses), Varoquaux found that studies with fewer subjects tended to report higher prediction accuracies (Varoquaux, 2018). Larger and more diverse samples are crucial to train properly evaluated models that can detect subtle patterns and generalise to the heterogeneity encountered in the clinical setting (Q. Ma et al., 2018;Traut et al., 2022). Given the evidence of latent factors shared across psychiatric conditions and CNVs, a logical next step is to develop ML models that can better exploit the available data by combining information across related categories.

MTL is an ML framework in which, rather than training a model on a single learning task (e.g., predicting ASD from rs-fMRI data), a model is trained on multiple related tasks concurrently; for example, predicting a diagnosis of ADHD as well as a diagnosis of ASD from resting-fMRI data (Huang et al., 2020). When the tasks are well grouped together, MTL can make better use of data by implicitly augmenting the data from each task with the data from the others, and the shared latent representation acts as a form of regularisation across tasks. Although there are still few examples in neuroimaging, MTL has been applied across target clinical variables using rs-fMRI data (Rahim et al., 2017) and combined imaging modalities (Zhang et al., 2012), across timepoints to predict disease progression using cortical surface data (Zhou et al., 2013), across individuals to perform brain decoding using fMRI data (Marquand et al., 2014;Rao et al., 2013), across fMRI task conditions to predict intelligence quotient (IQ) (Xiao et al., 2020), and across sites (Hu & Zeng, 2019;Q. Ma et al., 2018;Watanabe et al., 2014) and disease subtypes (X. Wang et al., 2015) to perform automatic diagnosis. Various deep learning architectures have also been applied to neuroimaging data using MTL (Dong et al., 2020;He et al., 2020;Liang et al., 2021;Ngo et al., 2020;Tabarestani et al., 2022;Yu et al., 2021). There are only two previous studies applying MTL across psychiatric conditions: the first (Huang et al., 2020) examined ASD and ADHD and the second (Huang et al., 2024) added SZ (prediction accuracy of 73.1, 72.7, and 84.9 respectively). In these studies, Huang and colleagues proposed the Multicluster Multigate Mixture of Experts (M-MMOE). The M-MMOE is a variant of the Multi-gate Mixture-of-Experts (MMOE) framework (J. Ma et al., 2018), in which MMOEs are combined across clusters of brain ROIs (seeSupplementary Materials section A.3for a more in depth description). While these results are in line with accuracies reported in the ML literature, they still fall short of being useful in clinical practice. Another limitation of these prior works is that they only combined a limited number of diagnostic categories in the MTL framework, in particular leaving out valuable but rare data on genetic risk for psychiatric conditions (CNVs) which might allow shared features that are subtle in psychiatric conditions to be learned more easily in highly impacted populations.

For this purpose, we used an rs-fMRI dataset consisting of subjects diagnosed with 7 CNVs and 4 psychiatric disorders, including a total of 2872 participants and 53 sites of data collection. We included CNVs that were previously found to be associated with psychiatric disorders: DEL 1q21.1, DUP 1q21.1, DEL 16p11.2, DUP16p11.2, DEL 22q11.2, DUP 22q11.2, and DEL 15q11.2 (Collins et al., 2022;Davies et al., 2020;Jønch et al., 2019;Marshall et al., 2017;Moreau et al., 2023;Sanders et al., 2015). In particular, DEL 22q11.2 and DEL 16p11.2 are rare examples of heritable (non de-novo) CNVs with severe clinical manifestations. Both variants have been found to have large clinical effect sizes (Crawford et al., 2019;Jonas et al., 2014;Moreau et al., 2023;Rees & Kirov, 2021;Willsey et al., 2022). DEL 22q11.2 is the biggest known risk factor for SZ: 30% of carriers will develop the condition in their lifetime (Marshall et al., 2017) and its diagnosis also carries an elevated risk for ASD (32 times higher than the general population). DEL 16p11.2 is associated with ASD, as well as with ADHD (Moreno-De-Luca et al., 2013;Niarchou et al., 2019;Sanders et al., 2015). We included common psychiatric disorders, which have also been found to be associated with CNVs: autism spectrum disorder (ASD) associated with 16 different CNVs, schizophrenia (SZ) associated with 14 different CNVs (Marshall et al., 2017;Rees and Kirov, 2021;Sanders et al., 2019;Satterstrom et al., 2020), and Bipolar (BIP) disorder and Attention-Deficit/Hyperactivity Disorder (ADHD) which are less frequently associated (Rees and Kirov, 2021). The estimated prevalence of ASD is 1% of children worldwide (Zeidan et al., 2022), of ADHD is 2.5% of the general population (Simon et al., 2009), of BIP is 1% worldwide (Merikangas et al., 2011), and of SZ is 0.44% of the general population (Moreno-Küstner et al., 2018). Comorbidities between these conditions are extensive (Katzman et al., 2017;McElroy, 2004;Rösler et al., 2010;Sajatovic, 2005;Simonoff et al., 2008;Tsai & Rosenheck, 2013).

As a proof of concept, we first applied MTL in a context where there is clearly information shared across tasks: the same target (age or sex) is predicted at different sites of data collection where each site is treated as a task. Using the very large UK Biobank sample (30,185 subjects), we examined the impact of sample size on MTL accuracy. Next, we evaluated the potential benefits of MTL for prediction accuracy across the full set of psychiatric and genetic conditions in our dataset by treating each condition as a task. Finally, we explored the relationships between conditions by applying MTL to each pair using our standard model and several variant model architectures.

## 2 Materials and Methods

### 2.1 Ethics

The present secondary analysis project was approved by the research ethics review board at the Centre Hospitalier Universitaire Sainte-Justine.

### 2.2 Cohorts

The nine rs-fMRI datasets included four clinical CNV cohorts, five idiopathic neuropsychiatric datasets, and one very large sample of unselected individuals. A majority of the datasets are compiled from different sites of data collection and studies. In total, rs-fMRI data from 2872 individuals were included, who were either neurotypical control subjects, individuals diagnosed with one of 7 CNVs associated with psychiatric disorders (Moreau et al., 2023), or one of 4 psychiatric disorders (ASD, SZ, BIP, ADHD) (seeTable 1). The research ethics review boards of each relevant institution approved the study of the corresponding dataset.

**Table 1.** Demographics by condition.

	Condition	Total	N (F)	Age Mean	(SD)	FD Mean	(SD)	Sites	Dataset
**A**	DEL 15q11.2	103	(55)	64.29	(7.44)	0.19	(0.06)	3	UKBB
	Controls	103	(55)	62.65	(7.51)	0.19	(0.05)	3	
	DUP 16p11.2	35	(14)	34.15	(19.53)	0.21	(0.09)	6	MRG, SVIP, UKBB
	Controls	35	(14)	32.04	(20.34)	0.18	(0.06)	6	
	DUP 22q11.2	22	(12)	39.43	(23.49)	0.19	(0.09)	5	DEFINE, MRG, UCLA,
	Controls	22	(12)	38.61	(25.81)	0.17	(0.06)	5	UKBB
	DEL 1q21.1	25	(12)	44.40	(18.87)	0.18	(0.07)	6	DEFINE, MRG, SVIP,
	Controls	25	(12)	50.89	(14.69)	0.21	(0.08)	6	UKBB
	DUP 1q21.1	19	(13)	50.86	(19.35)	0.21	(0.08)	7	DEFINE, MRG, SVIP,
	Controls	19	(13)	51.40	(22.31)	0.17	(0.04)	7	UKBB
	DEL 16p11.2	32	(13)	21.74	(20.14)	0.22	(0.09)	5	DEFINE, MRG, SVIP,
	Controls	32	(13)	31.64	(20.15)	0.19	(0.07)	5	UKBB
	DEL 22q11.2	43	(19)	16.86	(6.95)	0.18	(0.07)	1	UCLA
	Controls	43	(22)	13.00	(4.61)	0.14	(0.04)	1	
**B**	ADHD	223	(66)	14.71	(9.47)	0.15	(0.04)	7	ADHD-200, CNP
	Controls	353	183	17.68	(10.63)	0.14	(0.04)	7	
	ASD	472	(0)	14.71	(5.88)	0.17	(0.05)	28	ABIDE1, ABIDE2
	Controls	471	(0)	15.32	(6.58)	0.16	(0.05)	28	
	SZ	283	(73)	33.90	(9.22)	0.17	(0.06)	12	Orban, CNP
	Controls	355	(113)	31.85	(9.33)	0.14	(0.05)	12	
	BIP	44	(20)	35.02	(8.95)	0.17	(0.07)	2	CNP
	Controls	113	(52)	30.88	(8.59)	0.14	(0.04)	2	

A) Psychiatric CNVs, B) Psychiatric Conditions. The first two columns are the number of total subjects, and of female subjects (in parentheses). The intermediate columns show the mean age and framewise displacement (FD) (a measure of head motion, with standard deviation (in parentheses)). The final column shows the number of scanning sites contributing to the dataset. SeeSection 2.2for dataset abbreviation definitions.

#### 2.2.1 Clinical genetic datasets

Participants in the four clinical genetic rs-fMRI datasets were recruited for scanning based on the presence of a CNV regardless of the presentation of symptoms, along with matched control subjects. These four clinical CNV datasets included the Simons Variation in Individuals Project (SVIP) (Simons Vip Consortium, 2012), the DEFINE Neuropsychiatric-CNVs Project (DEFINE) (Cardiff, United Kingdom) (Drakesmith et al., 2019), the University of California, Los Angeles 22q11.2 CNV project (UCLA) (Jalbrzikowski et al., 2022;Lin et al., 2017;Schleifer et al., 2024), and the unpublished Montreal rare genomic disorder family project (MRG) (MRG, Canada).

#### 2.2.2 Psychiatric conditions cohorts

We included 5 psychiatric rs-fMRI datasets: Autism Brain Imaging Data Exchange 1 (ABIDE1) (Di Martino et al., 2014), Autism Brain Imaging Data Exchange 2 (ABIDE2) (Di Martino et al., 2017), ADHD-200 (ADHD-200 Consortium, 2012), Consortium for Neuropsychiatric Phenomics (CNP) (Poldrack et al., 2016), and an aggregate dataset of 10 SZ studies (Orban) (Moreau et al., 2020;Orban et al., 2017). These studies provided data for individuals with ASD, ADHD, SZ, BIP, and matched control subjects.

#### 2.2.3 Unselected population

CNV carriers with available rs-fMRI data were identified in the UK Biobank (UKBB) (Sudlow et al., 2015) using PennCNV (K. Wang et al., 2007) and QuantiSNP (Colella et al., 2007) following previously published methods (Huguet et al., 2021;Martineau et al., 2019). The DNA was extracted from blood samples; the Affymetrix arrays were utilised, sharing common probes between them, with a scale of 50 k on the UK BiLEVE Array and 450 k on the UK Biobank Axiom Array (Wain et al., 2015).

### 2.3 rs-fMRI preprocessing

All datasets were preprocessed using the same parameters of NIAK (Bellec et al., 2012). The three first volumes of each run were suppressed to allow the magnetisation to reach equilibrium. Each fMRI dataset was corrected for inter-slice difference in acquisition time, and the parameters of rigid-body motion were estimated for each time frame. Rigid-body motion was estimated within as well as between runs. The median volume of one selected fMRI run for each subject was coregistered with a T1 individual scan, which was itself non-linearly transformed to the Montreal Neurological Institute (MNI) template (Fonov et al., 2011). The rigid-body transform, fMRI-to-T1 transform, and T1-to-stereotaxic transform were all combined, and the functional volumes were resampled in the MNI space at a 3 mm isotropic resolution. The “scrubbing” method (Power et al., 2012) was used to remove the volumes with excessive motion (frame displacement greater than 0.5). The following nuisance parameters were regressed out from the time series at each voxel: slow time drifts (basis of discrete cosines with a 0.01 Hz high-pass cut-off), average signals in conservative masks of the white matter and the lateral ventricles, as well as the first principal components (95% energy) of the six rigid-body motion parameters and their squares (Giove et al., 2009;Lund et al., 2006). The fMRI volumes were finally spatially smoothed with a 6 mm isotropic Gaussian blurring kernel. A more detailed description of the pipeline can be found on the NIAK website. Preprocessed data were visually controlled for the quality of the co-registration, head motion, and related artefacts.

### 2.4 Computing connectomes

We used the Multiresolution Intrinsic Segmentation Template (MIST) brain parcellation (S. Urchs et al., 2017) to segment the brain into 64 regions. This functional brain parcellation was found to have excellent performance in several ML benchmarks on either functional or structural brain imaging (Dadi et al., 2020;Hahn et al., 2022;Mellema et al., 2022). We chose the 64 parcel atlas of the MIST parcellation because this range of network resolution was found to be sensitive to changes in functional connectivity (FC) in psychiatric disorders, both using ML (Dadi et al., 2020;Hahn et al., 2022;Mellema et al., 2022) as well as classical mass univariate regression (Bellec et al., 2015). FC between any two regions was defined as the Fisher z-transformed Pearson’s correlation between the average time series of each region, while within region connectivity is the Fisher z-transformed average of Pearson’s correlation between any pair of distinct voxels within the region. Each connectome consisted of 2080 values: (63*64)/2 = 2016 region-to-region connections plus 64 within-region connectivity values.

### 2.5 Multi-task learning

We explored using MTL to predict age and sex across sites, and to perform automatic diagnosis across conditions from connectomes using shared bottom neural network models. For age and sex prediction each site is treated as a task, and for automatic diagnosis each condition is treated as a task. In shared bottom models (often called hard parameter sharing), the first layers of the network are common to all tasks after which the model branches into a series of task-specific heads (seeFig. 1B). We chose to implement this form of MTL rather than various soft-parameter sharing schemes, in which partially or entirely parallel networks have parameters regularised jointly, first because it is a very commonly used approach and, second, because the reduction in parameters and hence capacity is well suited to our high-dimensional data. We used a simple Multi-Layer Perceptron (MLP) architecture throughout our experiments, with either two outputs for binary classification tasks (MLPconn) or a single output for regression (MLPconn_reg), and added variants (described below) to explore the relationships between tasks. We also explored different parameters of the MLPconn and MLPconn_reg architectures as a sensitivity analysis; seeSupplementary Materials section A.9. All models were implemented in Pytorch (Paszke et al., 2019), and the code for MTL was written using Snorkel (Ratner et al., 2017) as a reference.

**Fig. 1.**
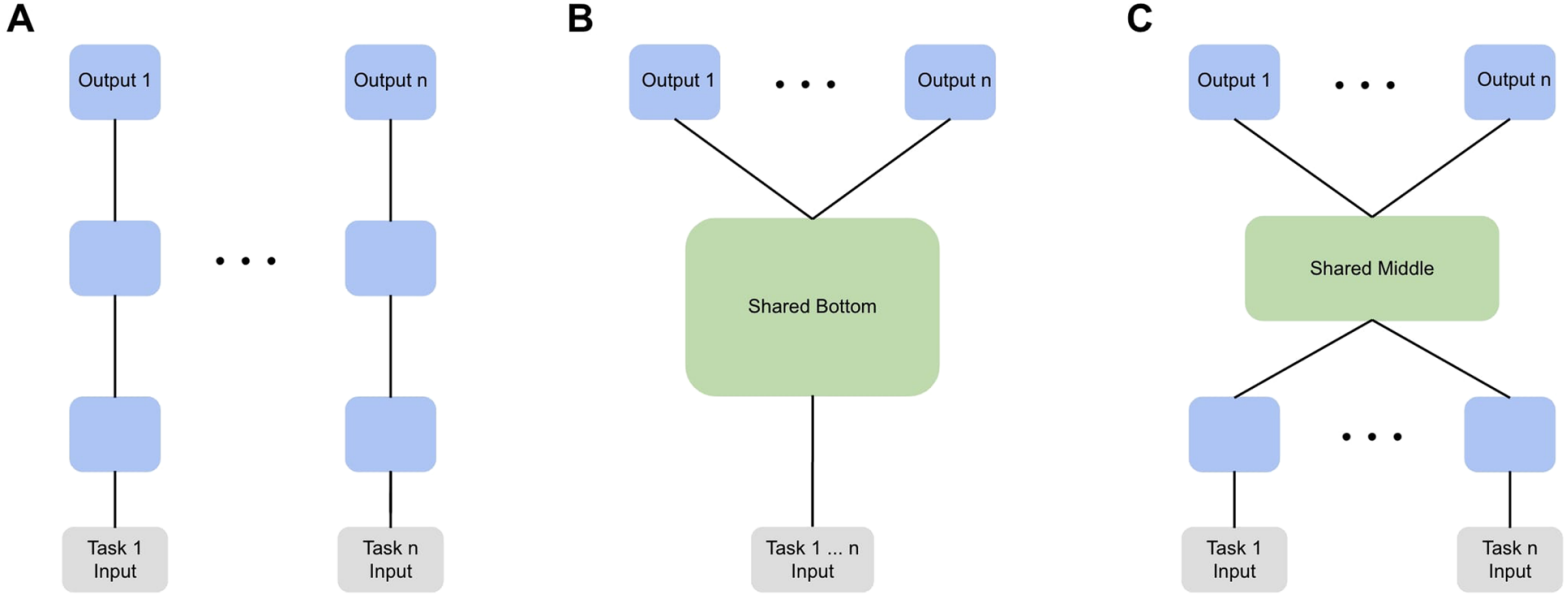
(A) Single-task learning, (B) Shared bottom model, (C) Shared middle model.

The MLPconn model is an MLP with the following configuration: 2080-256-64-2. The input to the networks is a 1 × 2080 vector consisting of the upper triangular values of the symmetric connectome matrix, which is passed through two shared hidden layers with 256 and 64 units and finally to a task-specific output layer of 2 units for binary classification. Batch normalisation (Ioffe & Szegedy, 2015) is applied after each layer. In the single-task setting, all the layers of the network are specific to the given task.

The MLPconn_reg model is the same as MLPconn, but with the output layer modified for regression so that the configuration becomes: 2080-256-64-1.

The MLPconcat model is exactly the same as the MLPconn model, with the input layer adapted to take as input a concatenation of the upper triangular 1 x 2080 connectome vector with the 1 x 58 confounds vector (age, head motion, global signal, scanning site, and sex with categorical confounds one hot encoded). The result is an MLP model with configuration: 2183-256-64-2.

The MLPconn_deeper model is a version of the MLPconn model with two additional layers of width 64: one in the shared part of the model and another in the task specific part. The resulting configuration is 2080-256-64-64-64-2. The input to the model is the connectome vector.

The convolutional neural network (CNN) model is adapted fromLeming and Suckling (2021). The input to the network is the upper triangle of the symmetric connectome matrix (2080 values), randomly permuted and formatted into a 40 x 52 matrix. The shared part of the model consists of a first convolution layer with 256 filters of shape 1 x 40 x 1, followed by two dense layers of 64 hidden units. The task-specific output layer has 2 units for binary classification. Batch normalisation (Ioffe & Szegedy, 2015) is applied after each layer. This implementation of a CNN is not designed to take into account spatial or functional relationships between regions; seeSupplementary Materials section A.10for a more in-depth discussion and comparison with other architectures.

The shared middle (SM) model is a variant of the shared bottom framework, in which each task has its own specific input layer, followed by layers shared across tasks, and finally the task-specific head (seeFig. 1C). Specifically, it has the configuration 2080-256-256-64-2: the input to the model is the upper triangular 1 x 2080 connectome vector, the first layer with 256 units is task specific, followed by two shared layers with 256 and 64 units, then a task-specific head with two output units. Batch normalisation (Ioffe & Szegedy, 2015) is applied after each layer.

### 2.6 Training

We trained the MTL models as follows for each epoch: first, the batches of data are pooled across tasks and shuffled (seeFig. 2); next, each batch is passed through the path it is associated with (through task-specific and shared layers), the loss is calculated and back propagated through the same layers; and finally, the gradients are clipped to have a maximum magnitude of 1. In the single-task setting, the training followed the same procedure except that the batches of data were not pooled across tasks and were fed through a fully independent network. We used a small batch size (8) since we included small datasets, and models were trained for 100 epochs, roughly 50 epochs past observing plateaus in the single-task setting. We used the Adam optimizer (Kingma & Ba, 2014), Leaky ReLUs as an activation function, and dropout regularisation (Srivastava et al., 2014) with the default parameters (Paszke et al., 2019). The binary classification tasks were trained using the cross-entropy loss after applying the softmax function, and the regression tasks with the mean squared error (MSE) (average of the squared differences between the predicted and actual scores). Classification tasks were additionally scored using prediction accuracy (number of correctly classified subjects divided by the total number of subjects), as well as the Area Under the Receiver Operating Characteristic (AUC) and F1-scores (seeSupplementary Materials section A.5).

**Fig. 2.**
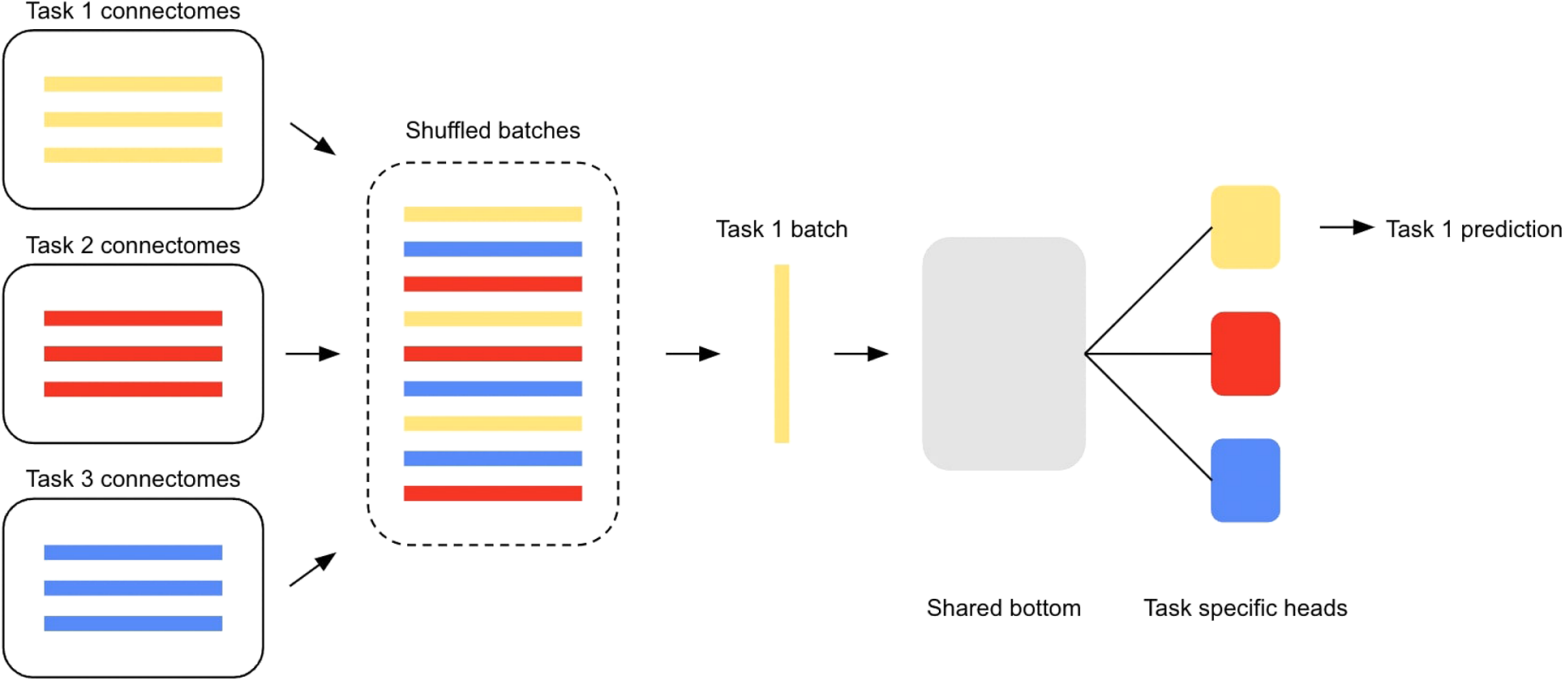
The training process for MTL using the shared bottom model illustrated for three tasks. Connectomes from each task (conditions or sites of data collection) are shuffled and then fed to the model. Batches are fed to the model one at a time, and a batch from a given task is fed to its respective task-specific head.

### 2.7 Predicting sex and age

Before we delved into the complexity of MTL across CNVs and psychiatric conditions, we evaluated the benefit of using multi-task learning where heterogeneity between tasks is only due to sites. We treated each site of data collection as a task and predicted the same target (either sex or age) across them; the prediction was performed from the connectomes alone and evaluated using 5-fold cross-validation. To predict sex, the MLPconn model was used first in the single-task setting to establish a baseline and then in the multi-task setting with all sites pooled together. For predicting age, the MLPconn_reg model was used again in single task and then multi-task across sites.

We first applied this approach using only the three sites from UKBB, since they have sample sizes (4569, 7943 and 17673) that are large enough that we could systematically quantify both the impact of sample size (similar to the experiments ofSchulz et al. (2020)) and the impact of MTL. Here, we looked at two scenarios: in the first, we sampled K subjects from each site and compared the prediction of single-task models to a multi-task model across the three sites, effectively tripling the number of subjects available to the multi-task model. In the second, we compared a multi-task model across a sample of K subjects from each site to a single-task model (on the largest site) with 3 x K subjects, effectively keeping the sample size the same between the multi-task and single-task model.

Next, we used the control subjects from each site of data collection in our sample that had at least 30 such participants. We subsampled 50 subjects from each of the very large UKBB datasets (sample sizes of 4569, 7943 and 17673) to place them within the range of the other sites. For the sex prediction task, we excluded sites that had an insufficient number of female subjects (NYU, SZ1, SZ2, USM) (seeTable 2and figures inSupplementary Materials section A.7.1). For both the sex and age prediction, we performed ablation studies in which each site was dropped from the set of tasks as a sensitivity analysis; seeSupplementary Materials section A.8.

**Table 2.** Demographics of control subjects by scanning site.

Site	N	Female	%	Age	Dataset
ADHD1	54	35	65	10.93 (1.63)	ADHD-200
ADHD3	56	26	46	10.22 (1.27)	ADHD-200
ADHD5	77	39	51	12.25 (3.12)	ADHD-200
ADHD6	39	18	46	9.30 (1.25)	ADHD-200
HSJ	39	25	64	34.03 (16.10)	MRG
NYU	66	0	0	15.68 (6.22)	ABIDE1
SZ1	42	3	7	34.05 (10.90)	Orban
SZ2	41	2	5	31.54 (8.68)	Orban
SZ3	31	15	48	31.00 (8.19)	Orban
SZ6	35	12	34	29.03 (8.48)	Orban
Svip1	48	18	38	28.25 (16.56)	SVIP
Svip2	36	17	47	24.62 (12.44)	SVIP
UCLA_CB	43	22	51	13.00 (4.62)	UCLA
UCLA_DS1	94	43	46	31.10 (8.72)	CNP
UKBB11025	17673	9342	53	63.43 (7.50)	UKBB
UKBB11026	4569	2504	55	65.52 (7.54)	UKBB
UKBB11027	7943	4414	56	64.80 (7.45)	UKBB
USM	30	0	0	20.76 (7.21)	ABIDE1

Number of total and female subjects, percentage female by site, and mean age in years with standard deviation in parentheses.

### 2.8 Class imbalances

The CNV datasets have major class imbalance, with far more controls than case subjects. Major class imbalances are problematic for predictive modelling; therefore for this context, we created datasets using the General Class Balancer algorithm (Leming et al., 2020) which were balanced exactly with respect to diagnostic classes and approximately with respect to the distribution of confounding variables inside each diagnostic class (age, head motion, global signal, scanning site, and sex). The General Class Balancer algorithm exactly matches categorical variables, while continuous confounds are matched by recursively quantising into smaller and smaller bins until subjects can be matched across bins while the distributions of the confound between classes are not found to be statistically different using a Mann-Whitney U-test. For most of the CNVs, we applied General Class Balancer with no modifications. The General Class Balancer algorithm repeatedly failed to find a match for a specific subject with DUP 16p11.2 when launched with different random seeds; we hand-selected the closest matching control for this subject. The DEL 22q11.2 dataset was collected entirely from a single site, and participants were recruited in a balanced design; in this case, we used all the subjects available without applying General Class Balancer. For the psychiatric conditions, the sample size was markedly larger than with CNVs, and class imbalance was also less severe. We thus used all the available cases and controls from each study, without application of General Class Balancer. The distribution of confounding variables for each of the balanced datasets is presented inSupplementary Materials section A.7.2.

### 2.9 Predicting CNVs and psychiatric conditions

In order to establish a baseline with which to compare our MTL results, we first performed automatic diagnosis in the single-task setting (seeFig. 1A), in which each task is learned by an independent model. In addition to the MLPconn model (described above), we evaluated three well-performing (Dadi et al., 2019) ML algorithms implemented in scikit-learn (Pedregosa et al., 2011): Support Vector Classifier (SVC) (linear kernel, C = 100, and*L*_2_penalty), Logistic Regression (LR) (*L*_2_penalty), and Ridge Regression (Ridge). Next, we trained the MLPconn model in the MTL setting across all 7 CNVs and 4 psychiatric conditions. The models were evaluated using intra-site cross-validation (Orban et al., 2018), in which the model is exposed to identical sites of data collection during training and testing to account for site effects. Specifically, five non-overlapping folds of training and test groups are built for each dataset such that they have roughly the same proportion of cases and controls from each site. Both the training and test groups feature every available site at each fold. The reported accuracy is the average of the model’s performance across all folds.

### 2.10 Study of task relationships

We added a fine-grained analysis to explore the task relationships between the 7 CNVs and 4 psychiatric conditions by training the conditions together pairwise using our primary model (MLPconn) and four variations that explored different model capacity (MLPconn_deeper), input data (MLPconcat), encoder type (CNN), and parameter sharing scheme (SM). This framework allowed us to characterise whether relationships between tasks behaved differently depending on the context. Each model was first evaluated in the single-task setting to establish a baseline.

## 3 Results

### 3.1 Multi-task learning of sex and age prediction across sites

#### 3.1.1 Multi-task learning across sites improves sex prediction in UK Biobank

We first evaluated the benefit of using MTL in a simple binary classification setting where heterogeneity between tasks is only due to sites. We treated each site of data collection as a task and predicted sex across them. We applied this approach using only the three large sites (n = 4569, 7943, and 17673) from UKBB in order to compare single-task learning and MTL at a range of sample sizes. First, we sampled K subjects from each site and compared the prediction of single-task models to a multi-task model across the three sites (seeFig. 3A). There was a clear gain in performance for MTL in this setting, which could be observed for all sample sizes, although becoming small for N > 4000. However, in this setup, the MTL model effectively has access to triple the sample size which in and of itself improves prediction; therefore, we also compared an MTL model across a sample of K subjects from each site to a single-task model (only on the largest site which possessed sufficient subjects) with 3 x K subjects, effectively keeping the sample size the same across settings (seeFig. 3B). For large sample sizes (N > 7500), multi-task learning out-performed single-task learning, meaning that combining heterogeneous data intelligently can actually improve the quality of prediction. Overall, multi-task learning across sites seems to be beneficial for sex prediction in the UK Biobank, although the largest gains are due to increased sample size.

**Fig. 3.**
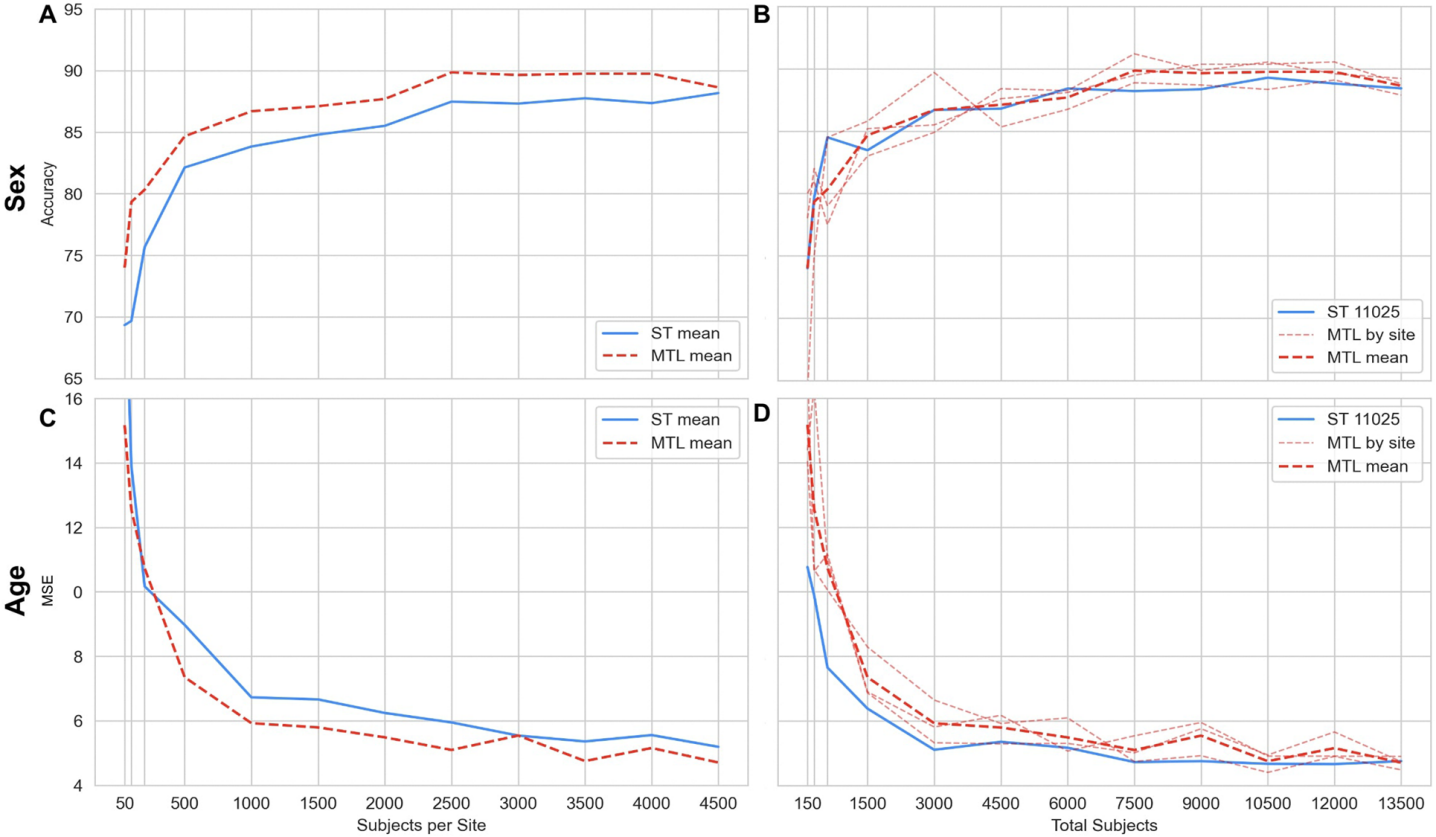
Prediction performance across sample sizes in the UKBB of single task (ST) versus multi-task learning (MTL) applied to a common target (age or sex) across sites. In the first setting (A & C), we kept the same subjects across the ST & MTL model; that is, for a sample of K subjects per site and ST model, the MTL model has access to 3 x K subjects in total. In the second setting (B & D), we aligned the sample size across models by comparing an MTL model with K subjects (across 3 sites) to an ST model with K subjects (using only the largest UKBB site where this was possible). (A & C) Mean accuracy of sex prediction (A) or Mean Squared Error (MSE) of age prediction (C) across sites for ST and MTL models. (B & D) Accuracy of sex prediction (B) or MSE of age prediction (D); mean performance across sites using MTL model (red dashed line), performance of MTL model at each individual site (pale red dashed line), and of ST model on UKBB site 11025 (blue solid line).

#### 3.1.2 Multi-task learning across sites improves age prediction in UK Biobank only when increasing sample size through site pooling

We repeated the previous experiment using the three large UKBB sites, but this time using age as a prediction target, which is continuous and more challenging. In the first setting in which we sampled K subjects from each site for the single-task models and used the same subjects (3 x K) for the MTL model (seeFig. 3C), there was an advantage for MTL, but only for a relatively large sample size (N > 500 per site, 1500 total). For smaller sample sizes, single-task and multi-task models had very similar performance. In the second setting in which we kept sample size fixed by comparing an MTL model across a sample of K subjects from each site to a single-task model with 3 x K subjects (seeFig. 3D), it became apparent that MTL models underperformed compared to the single-task model, with the gap in performance decreasing with sample size, and becoming very small for N > 10 k. Overall, MTL across sites seemed to benefit age prediction across the UKBB sites but only to the extent that it enables to increase sample size through site pooling.

#### 3.1.3 Multi-task learning across sites improves sex prediction across cohorts

Next, we used the control subjects from each site of data collection in our sample that had at least 30 participants and predicted sex across them using the MLPconn model, excluding sites that had an insufficient number of female subjects (NYU, SZ1, SZ2, USM). We subsampled 50 subjects from each of the very large UKBB datasets (n = 4569, 7943, and 17673) to place them within the range of the other sites. In this setting, MTL effectively pools subjects for a larger sample size at the price of greater heterogeneity. Prediction accuracy improved for MTL in a majority of sites (10 out of 14) (seeFig. 4). The mean accuracy in the multi-task setting (62.4) outperformed that of the single task (60.0), although not significantly (Wilcoxon’s signed-rank test) and with larger standard deviation (13.6 vs. 12.8) (seeSupplementary Materials section A.4–Figure 13for the distribution across folds). Overall, MTL across heterogeneous sites of data collection benefitted accuracy for sex prediction.

**Fig. 4.**
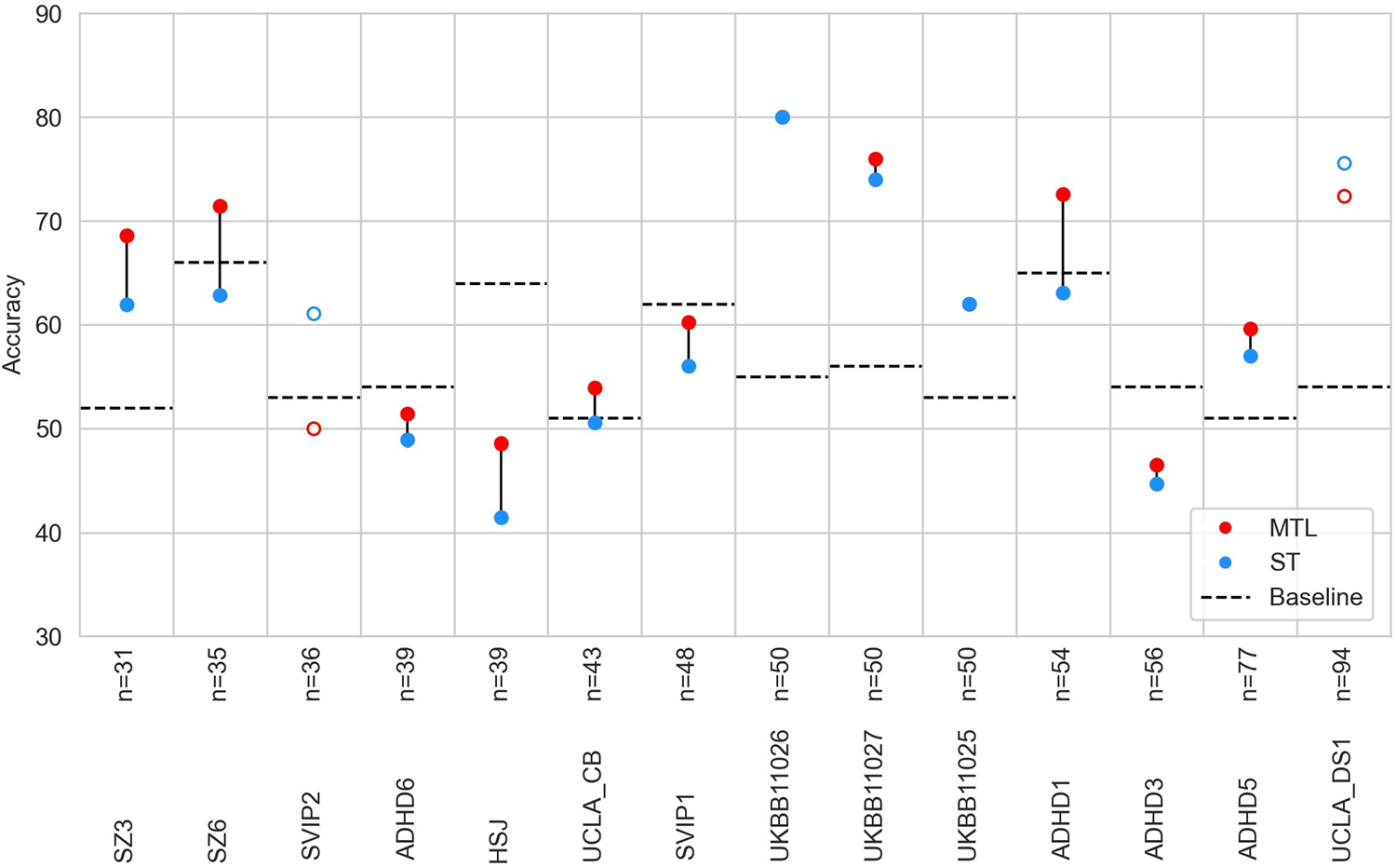
Accuracy of sex prediction using single (ST) versus multi-task learning (MTL) in a varied collection of sites. The x axis represents different data collection sites included as prediction tasks. Sites are ranked by sample size, with the largest to the right. The y axis shows the accuracy of prediction, and chance level of prediction is indicated by a black dashed line. For each task, the red point shows prediction using the MLPconn architecture in MTL, and the blue point shows prediction on the task trained independently using the MLPconn architecture. Where the red point appears missing, the accuracy values for the two models are so close that the points are overlapped. If the MTL prediction outperformed the ST, points were filled and connected by a line, and otherwise they were not.

#### 3.1.4 Multi-task learning across sites improves age prediction in a varied collection of samples

Next, we predicted age across each site of data collection (control subjects only) in our sample that had at least 30 participants using the MLPconn_reg model, again subsampling 50 subjects from each of the very large UKBB datasets. Each site of data collection consisted of subjects with markedly different age ranges (seeTable 2), so we expected this objective to be more difficult than sex prediction since MTL in this setting essentially works as a tradeoff between effectively increasing sample size at the cost of increased heterogeneity. Prediction improved for a large majority of sites (14 out of 18) (seeFig. 5). The mean loss in the multi-task setting (8.9 years^2^) significantly outperformed (Wilcoxon’s signed-rank test p < 2e-16) that of the single task (12.2 years^2^), but with a larger standard deviation (6.0 vs. 4.4) (seeSupplementary Materials section A.4–Figure 14for the distribution across folds). Overall, MTL benefitted prediction, even when the target of prediction was heterogeneously distributed across sites.

**Fig. 5.**
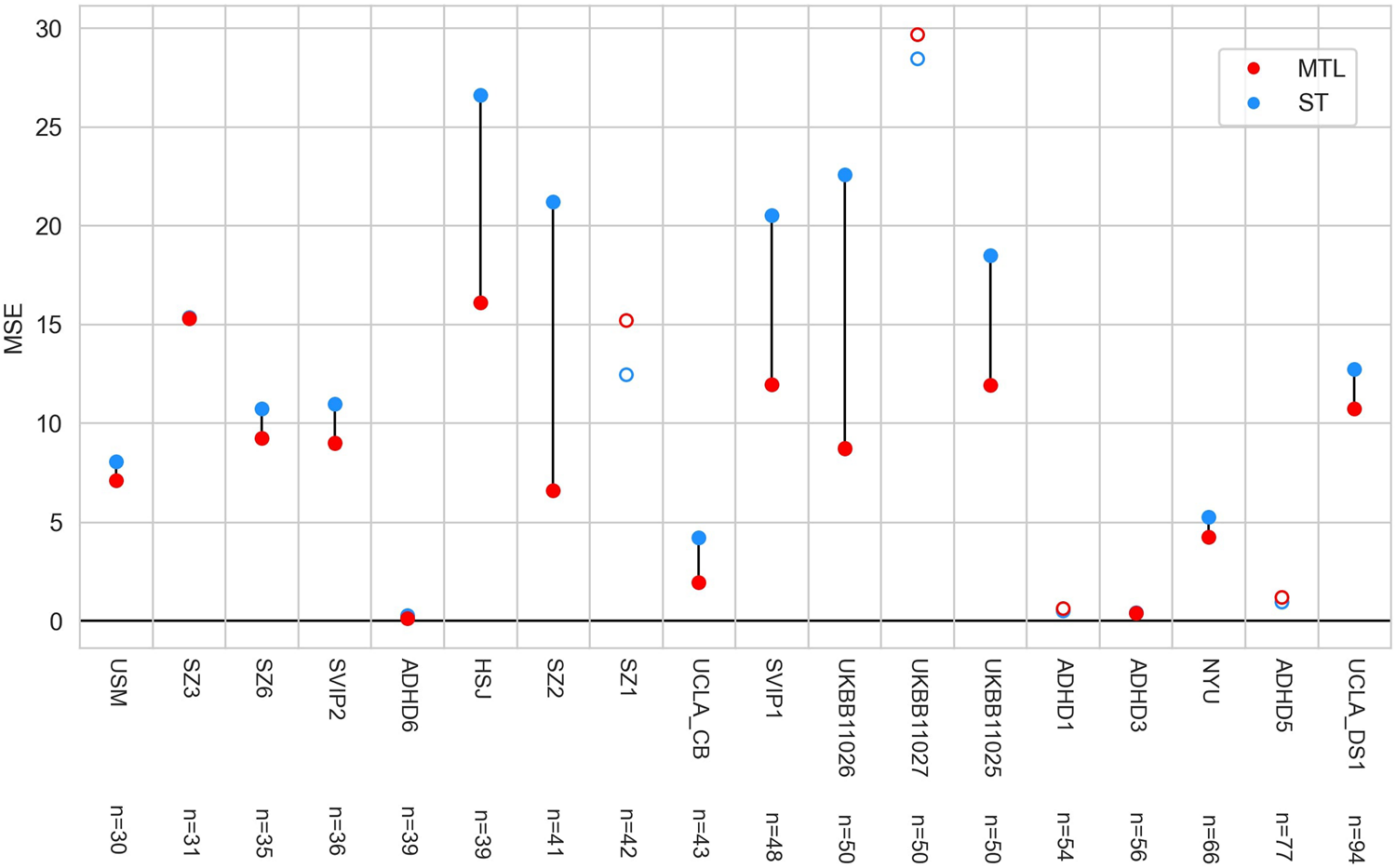
Mean Squared Error (MSE) of age prediction using single (ST) versus multi-task learning (MTL) in a varied collection of samples. The x axis represents different sites of data collection included as prediction tasks, and sites are ranked by sample size, with the largest to the right. The y axis shows the prediction error. For each task, the red point shows prediction using the MLPconn_reg architecture in multi-task learning and the blue point shows prediction on the task trained independently using the MLPconn_reg architecture. Where the blue point appears missing, the accuracy values for the two models are so close that the points are overlapped. If the MTL prediction achieved lower loss than the ST, points were filled and connected by a line, and otherwise they were not.

### 3.2 Multi-task learning fails to improve automatic diagnosis across psychiatric conditions and genetic variants

In order to establish a baseline with which to compare our MTL results, we first performed automatic diagnosis in the single-task setting. In addition to the MLPconn model, we evaluated three ML algorithms: Support Vector Classifier (SVC), Logistic Regression (LR), and Ridge Regression (Ridge). DEL22q11.2 reached the highest accuracy, close to 90% with LR and Ridge, while several other conditions reached over 70% accuracy (SZ, BIP, DEL 16p11.2, DUP 16p11.2, DEL 1q21.1) (seeSupplementary Materials section A.1–Fig. 8). The other conditions were very challenging to predict, being near (or below) chance level. However, the prediction accuracy for CNVs broadly follows the trend of clinical effect size (Moreau et al., 2023) with CNVs with near chance level accuracy having small effect sizes. Overall, standard ML models seem capable of automatically diagnosing most of the CNVs and psychiatric conditions. Next, we aimed to improve the automatic diagnosis of the 7 CNVs and 4 psychiatric conditions by leveraging shared information in datasets with limited sample size using a lightweight MTL framework to effectively increase the sample size available to the model. We trained the MLPconn model across all conditions and compared the performance to the same model trained on each condition independently. MTL outperformed single-task learning in only 3 out of 11 conditions (seeFig. 6), and in the remaining cases performance accuracy actually decreased (seeSupplementary Materials section A.4–Figure 15for the distribution across folds).

**Fig. 6.**
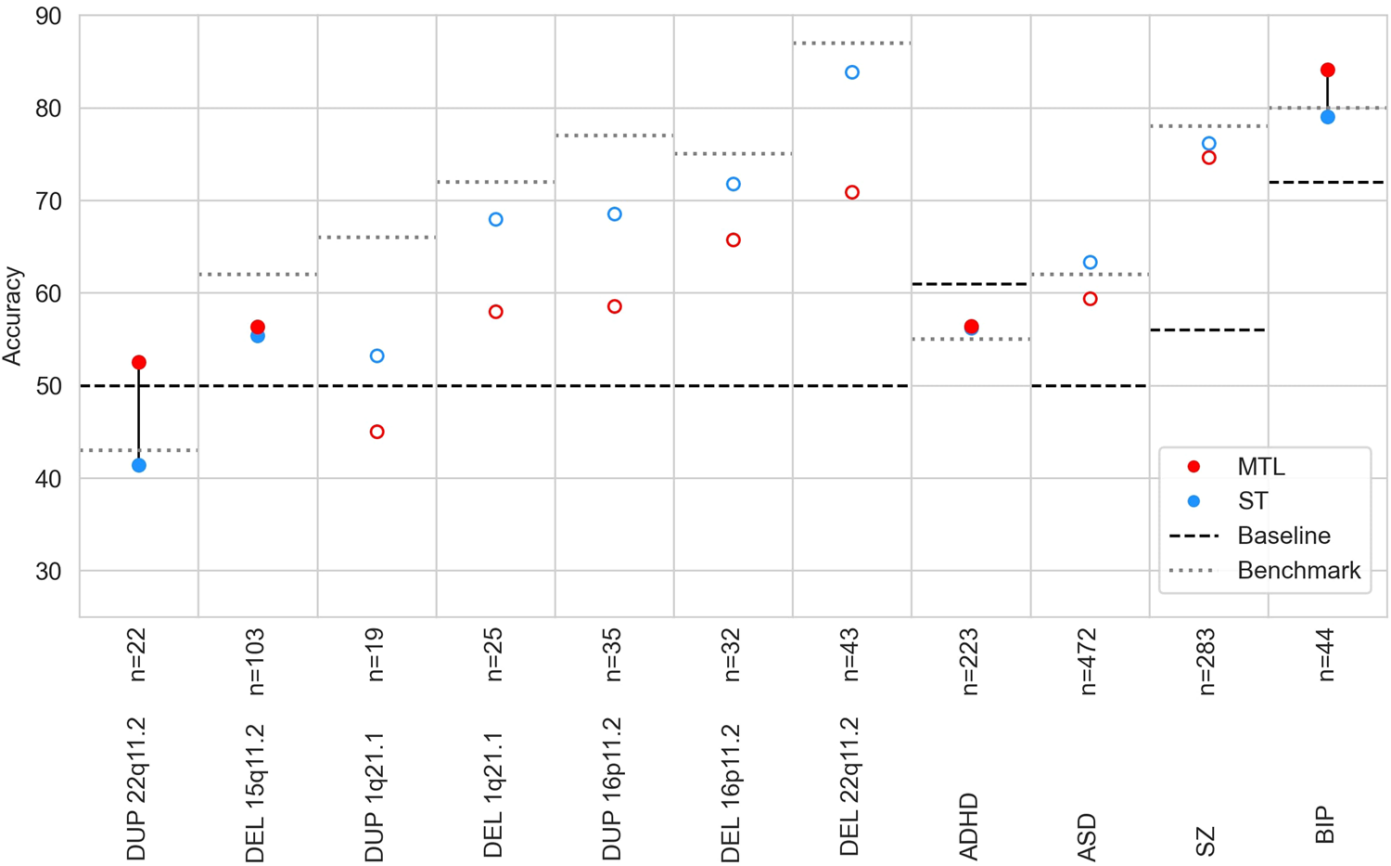
Accuracy of automated diagnosis using single (ST) versus multi-task learning (MTL). For each task, the red point shows prediction using the MLPconn architecture in MTL and the blue point shows prediction on the task trained independently using the MLPconn architecture. Where either the blue point appears missing, the accuracy values for the two models are so close that the points are overlapped. If the MTL prediction outperformed the ST, points are filled and connected by a line, and otherwise they are not. The x axis represents different conditions included as prediction tasks. The y axis shows the accuracy of prediction, chance level of prediction is indicated by a black dashed line, and the best accuracy obtained in the single-task learning benchmark (seeSupplementary Materials section A.1) is indicated by a grey dotted line.

### 3.3 Task relationships are dominated by sample size and accuracy of diagnosis in single-task setting

We aimed to disentangle the complexity of performing automatic diagnosis on the 7 CNVs and 4 psychiatric conditions in the MTL setting by training the conditions together pairwise to gain insight on the relationships between tasks. Using the MLPconn model, we found that the conditions with high accuracy in the single-task setting but small sample size (DEL 22q11.2 and DEL 16p11.2) suffered overall from being trained with a partner (seeFig. 7B). In contrast, regardless of accuracy in the single-task setting, the conditions with larger sample size (SZ, ADHD, ASD) were not impacted by their partner (seeFig. 7B). MTL appeared to benefit smaller sample size conditions with mid-range accuracy, but the results were not systematic. Certain pairs of conditions produced marked overall improvement in accuracy, notably DUP 22q11.2 + SZ and DUP 1q21.1 + DEL 1q21.1 (seeFig. 7C). We repeated the study using four different models (MLPconn_deeper, MLPconcat, CNN, and SM) (seeSupplementary Materials section A.2–Fig. 9), and correlated the matrix of change in accuracy from the single-task baseline for each with that of the MLPconn model. We found a range of correlations (r = 0.52, 0.26, 0.18, 0.19 respectively), which showed that the relationships between tasks were not stable across contexts. Overall, relationships between tasks appear to be dominated by the available sample size as well as the performance in the single-task setting, rather than reflecting potentially meaningful biological relationships.

**Fig. 7.**
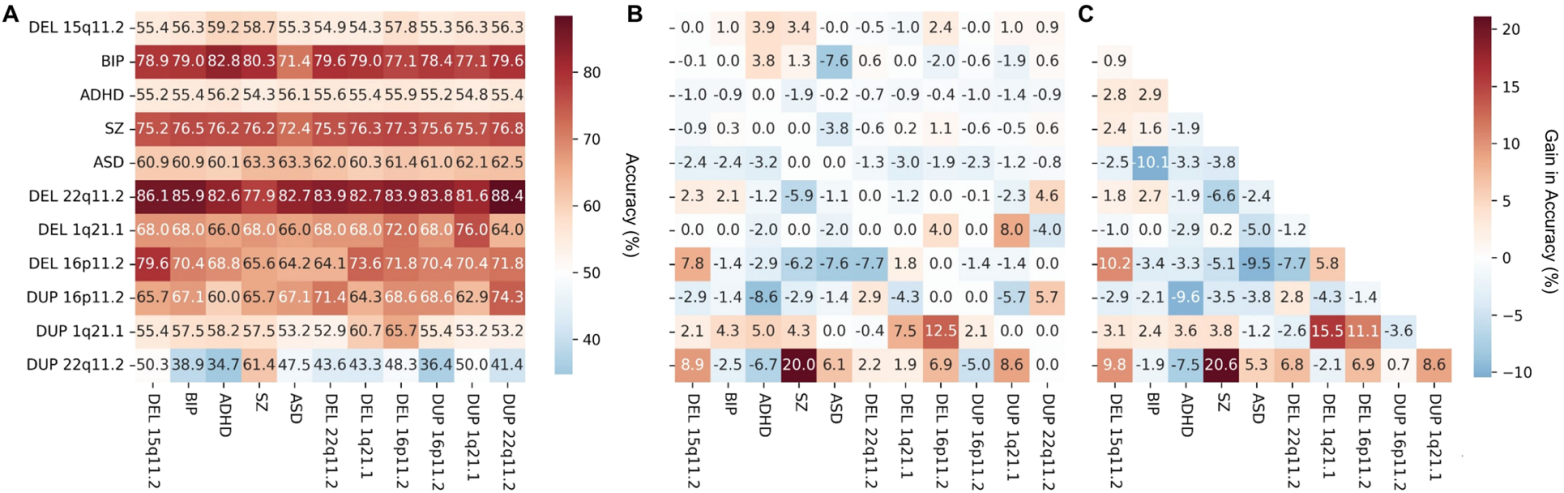
In each matrix, the i, jth entry in the matrix is accuracy of condition in row i trained with condition in column j using the MLPconn model to perform automatic diagnosis. (A): the matrix shows the raw accuracy achieved for each pair, the second matrix (B) represents the difference in accuracy from the single-task baseline, and the third (C) shows the overall gain relative to baseline for a pair (B + B^T^).

## 4 Discussion

Using MTL to predict a common target (sex or age) across sites in the UK Biobank at a range of sample sizes, we found that for large sample sizes MTL can improve prediction even when compared to single-task learning using the same number of subjects, but that MTL can be detrimental for small sample sizes (N < 500). When we applied MTL to predict a common target (sex or age) across the full sample of sites in our dataset, we found that it improved prediction. However, applying MTL across our diagnostic tasks (7 CNVs and 4 psychiatric conditions) was detrimental for performance overall. When we implemented MTL pairwise on the 7 CNVs and 4 psychiatric conditions in our dataset using our primary model and four variations, we found that the relationships between tasks were not stable across model architectures.

Repeating prediction across a range of sample sizes in the UK Biobank, we found that using MTL to predict a common target (sex or age) across data collection sites (3 x N subjects, N subjects per site from 3 sites) is not as strong as prediction at a single site with the same amount of data (3 x N subjects from 1 site), but is stronger in general than performing prediction at each site independently (N subjects from 1 site). These results are in line with findings bySchulz et al. (2020), who classified subjects into groups divided by sex and age using fMRI data and simple linear models in the UK Biobank. They found that prediction accuracy improved with increasing sample size, but did not investigate the effects of sites or MTL models. When predicting age, we saw that MTL did not necessarily improve prediction when sample sizes were small (N < 500).Standley et al. (2019)also examined the impact of sample size on MTL, comparing a shared bottom model trained on a large dataset to the same model trained on a subsample (5% of the original data). They found that while MTL was overwhelmingly beneficial for prediction using the full sample, it hurt prediction overall when less data were used to train the model. Essentially, MTL allows a shared model to access more data than independent models could, but the combined data naturally introduce heterogeneity. While there is a possibility to benefit from this as regularisation across tasks, there exists a regime with modest amounts of data where MTL can be detrimental for performance.

When we applied MTL to predict a common target (sex or age) across the full sample of sites in our dataset, we saw that performance was improved for a large majority of sites. While we expected that MTL would be beneficial for sex prediction, it was especially encouraging that prediction of age was improved since in light of the scaling experiment it was unclear if our sample sizes (N = 881 for MTL vs. N = 30–94 for single task) could benefit from MTL. The most notable difference with the UK biobank experiment was that we had far more sites of data collection (18 sites vs. 3 sites), and the sites were also more varied (retrospectively pooled across independent studies vs. harmonised acquisitions from a single study). Our results suggest that there might be some benefit not only from increasing sample size but also from combining diverse information, in line with previous work on SZ diagnosis (Orban et al., 2018). Several studies have also reported improved prediction when using MTL across sites as applied to automatic diagnosis of SZ (Hu & Zeng, 2019;Q. Ma et al., 2018) and ADHD (Watanabe et al., 2014). While these studies did not include scaling experiments, in each case MTL was an improvement over pooling heterogeneous data. MTL learning thus appears as a viable alternative to data harmonisation across sites (El-Gazzar et al., 2023;Roffet et al., 2022;Y.-W. Wang et al., 2023), with the potential to improve prediction accuracy in the presence of moderate heterogeneity.

Applying MTL across our diagnostic tasks (7 CNVs and 4 psychiatric conditions), we saw that it was detrimental for performance overall. One possible conclusion is that the tasks are too heterogeneous to benefit from being learned together, and that rather than using a combined model across tasks we should pursue an approach that emphasises finding homogeneous subtypes within conditions, as has been explored in the context of high precision modelling (S. G. W. Urchs et al., 2020). It is, however, illuminating to look at our UK biobank scaling experiment and note that the difference between the combined sample size using MTL (N = 2872) and the single-task sample sizes (N = 44–943) is not far from the threshold for MTL to consistently improve prediction of age (N > 500 single task, >1500 MTL). As joint diagnosis is a much more complex objective (seeSupplementary Materials section A.6for a more detailed analysis), it seems natural that it would require much more data to see a benefit from MTL. Although a speculation, our results thus suggest that the application of MTL to automated diagnosis across psychiatric conditions may be successful only if applied with over 500 patients per condition, that is, several thousand subjects in total. This parallels the conclusions ofMarek et al. (2022)who concluded that thousands of subjects are needed for reliable mass univariate brain/behaviour associations.

In the only comparable studies in the literature,Huang et al. (2020,^2024^) proposed a variant of the MMOE model (J. Ma et al., 2018) to perform joint diagnosis across psychiatric conditions. In their second study, their method showed marginal gains for each condition (N = 72 SZ, 358 ADHD, 505 ASD) relative to single-task learning, while a shared bottom model reduced prediction accuracy for ADHD and SZ. We implemented additional experiments (seeSupplementary Materials section A.3) to test if our results could be attributed to limitations of the shared bottom model, but found that it was not consistently outperformed by the MMOE. While there are surely improvements that can be made to the MTL framework, we emphasise that increasing the amount of data is a crucial aspect of future ML research in neuroimaging.

When we implemented MTL pairwise on the 7 CNVs and 4 psychiatric conditions in our dataset using our primary model and four variations, we found that the relationships between tasks were not stable across contexts. This is in line with findings byStandley et al. (2019), who examined the matrix of prediction performance for tasks trained pairwise using a shared bottom model in different contexts (varying sample size and model capacity) and found that the relationships between tasks were dependent on the setting. This is contrary to our hypothesis that our MTL framework could uncover potentially meaningful biological relationships across the 7 CNVs and 4 psychiatric conditions in our dataset, which we would expect to be stable across different model architectures.

An important limitation of this study is that the datasets we had access to carry current biases in psychiatric research. It is well known that certain conditions (particularly ASD, ADHD, and SZ) are underrepresented among females, which reflects differences in understanding and diagnosing as well as prevalence (Attoe & Climie, 2023;Bierer et al., 2022;X. Li et al., 2022;Loomes et al., 2017;Pedersen et al., 2022).

We examined the important concept of using MTL to take advantage of information shared across biologically related conditions, possibly allowing automated diagnosis of conditions for which there are small amounts of available data and using traits that are easily learned in highly impacted CNV populations that could also apply to related psychiatric conditions. Although small sample size was a limitation of this study that was clear from the outset, the idea that the ability of MTL to make more efficient use of data would make it applicable to small datasets was not supported in our results. We found that applying MTL across conditions has potential, and although clever approaches to modelling, such as self-supervised (Caro et al., 2023) or transfer (Mahamud et al., 2023;Raghav et al., 2023) learning could potentially overcome the limitation of sample size, our results clearly show that increasing the amount of data is a crucial factor for improving prediction performance.

## 5 Conclusion

In this paper, we examined the potential of MTL to combine multiple automatic diagnosis tasks in a large fMRI dataset compiled from multiple studies. In an initial proof of concept, we predicted a common target (sex or age) across sites of data collection, and showed that MTL can be beneficial for prediction accuracy, with the important caveat that benefits for age prediction only became apparent for large sample sizes (N > 500 per site). We then benchmarked diagnostic accuracy of 7 CNVs and 4 psychiatric conditions using common machine-learning methods, for each condition independently. None of the CNVs had previously been studied using machine learning, and prediction accuracy aligned with results from the literature otherwise. We then applied MTL to test if learning conditions with shared latent biological factors jointly could benefit prediction. Contrary to our hypothesis, we observed that MTL harmed prediction accuracy overall. We further explored the behaviour of MTL by applying the framework on each pair of tasks and found that the relationships between tasks were not stable across varied contexts, which was evidence against the possibility of these relationships reflecting latent factors with biological meaning. Our scaling experiment with UK biobank suggests that MTL may become beneficial for automated diagnosis across neurodevelopmental conditions, but this would likely require larger sample sizes than what could be assembled in this study.

## Supplementary Material

Supplementary Material

## Data Availability

We thank all of the families at the participating Simons Variation in Individuals Project (SVIP) sites, as well as the Simons VIP Consortium. We appreciate obtaining access to imaging and phenotypic data on the SFARI Base. Approved researchers can obtain the Simons VIP population dataset described in this study by applying athttps://base.sfari.org. We are grateful to all families who participated in the 16p11.2 European Consortium. Data from UK Biobank were downloaded under the application 40980 and can be accessed via their standard data access procedure (seehttp://www.ukbiobank.ac.uk/register-apply). UK Biobank CNVs were called using the pipeline developed in Jacquemont Lab and described inhttps://github.com/labjacquemont/MIND-GENESPARALLELCNV. The final CNV calls are available from UK Biobank returned datasets (Return ID: 3104,https://biobank.ndph.ox.ac.uk/ukb/dset.cgi?id=3104). ABIDE1, ABIDE2, COBRE, ADHD-200, CNP, and 16p11.2 SVIP data are publicly available at:http://fcon_1000.projects.nitrc.org/indi/abide/abide_I.html,http://fcon_1000.projects.nitrc.org/indi/abide/abide_II.html,http://schizconnect.org/queries/new,http://fcon_1000.projects.nitrc.org/indi/adhd200/,https://www.openfmri.org/dataset/ds000030/, andhttps://www.sfari.org/funded-project/simons-variation-in-individuals-project-simons-vip/. The 22q11.2 UCLA raw data are currently available by request from the principal investigator (CEB). Raw imaging data for the Montreal rare genomic disorder family dataset are currently available by request from the principal investigator (SJ). The Cardiff raw data are not publicly available yet; contact the principal investigator for further information (DEJL). All processed connectomes are available through a request to the corresponding author (AH). Codes for all analyses are available online through the GitHub platform:https://github.com/harveyaa/neuropsych_mtl.
