# Supplementary material for: Challenges in multi-task learning for fMRI-based diagnosis: Benefits for psychiatric conditions and CNVs would likely require thousands of patients
Source: Imaging Neurosci (Camb). 2024 Jul 26;2:imag-2-00222. doi: 10.1162/imag_a_00222 (PMC12290746; doi:10.1162/imag_a_00222)
Supplement: Supplementary Material [file imag_a_00222-supp.pdf]

Appendix A - Supplementary Materials

A.1 - Single Task Learning Benchmark - Conditions

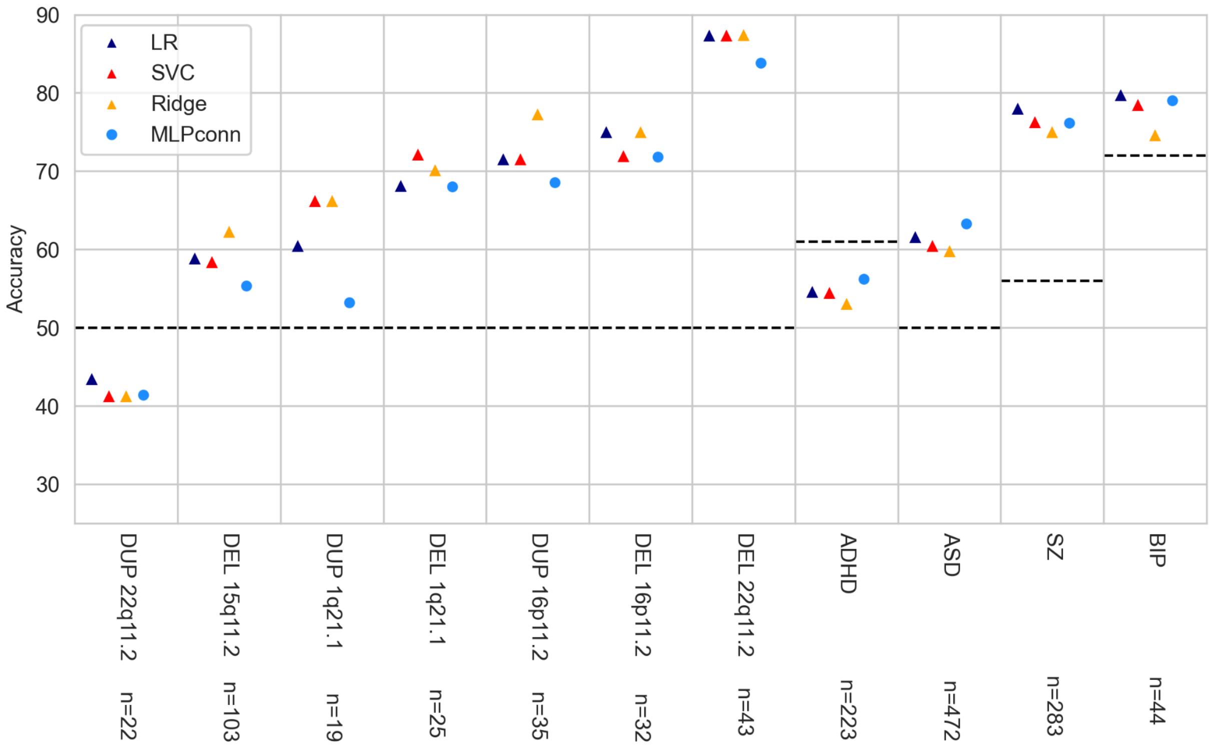

Figure 8 - Accuracy of automated diagnosis using single task learning. For each task, accuracy is shown for each of four models. LR: Logistic Regression, SVC: Support Vector Classifier, Ridge: Ridge Regression, and MLPconn. The x axis represents different conditions included as prediction tasks. The y axis shows the accuracy of prediction, chance level of prediction is indicated by a black dashed line.

A.2 - Study of Task Relationships using Variant Models

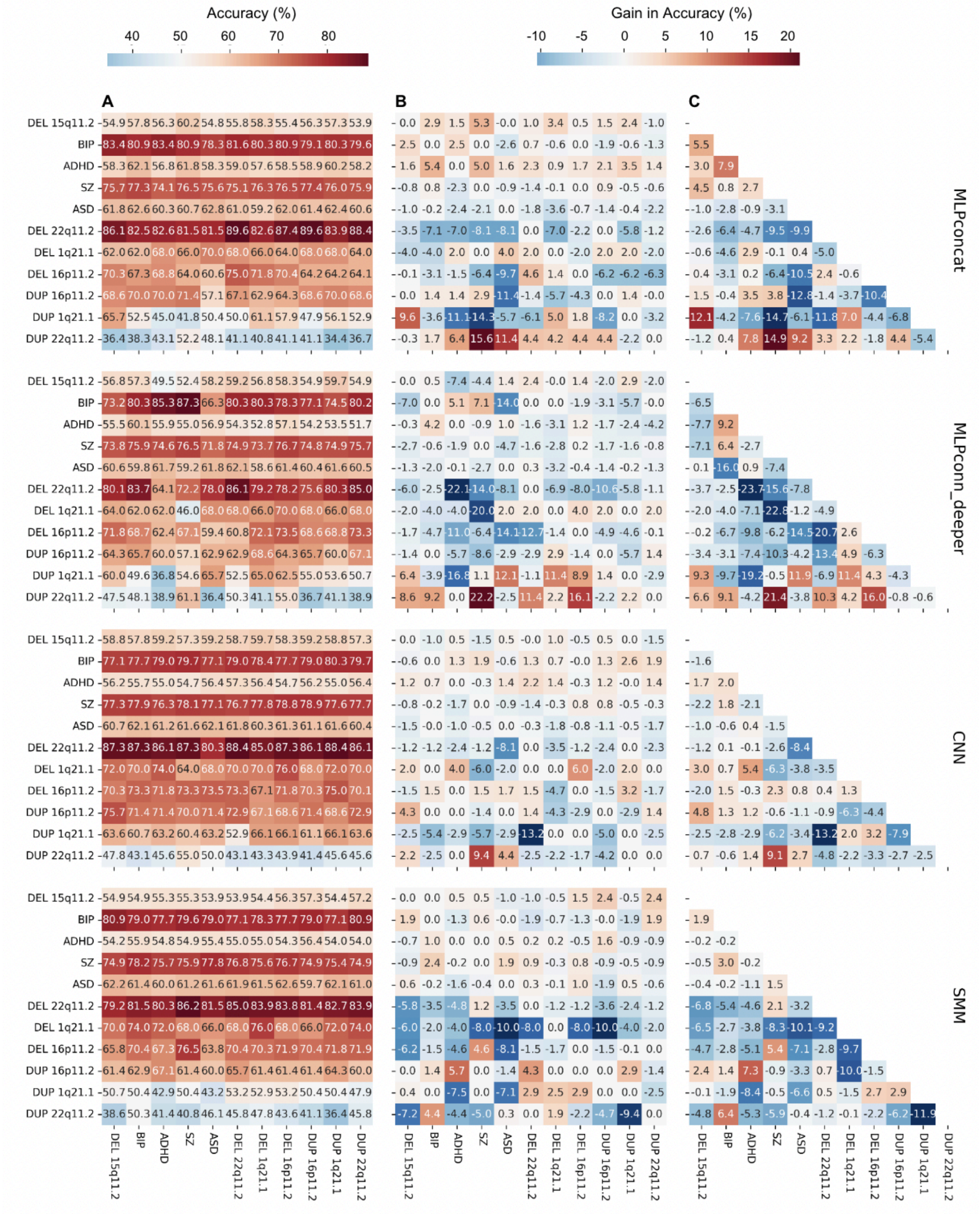

Figure 9 - In each matrix, the i,jth entry in the matrix is accuracy of condition in row i trained with condition in column j using the MLPconn model to perform automatic diagnosis. A: the matrix shows the raw accuracy achieved

for each pair, the second matrix B represents the difference in accuracy from the single-task baseline, and the third C shows the overall gain relative to baseline for a pair ($B + B^T$). Each row shows the results using the labelled model (MLPconcat, MLPconn_deeper, CNN, SMM), see the methods for details.

A.3 - Comparison with Huang and Colleagues

We implemented additional experiments to allow a closer comparison of our results to the only studies in the literature to apply MTL across conditions (Huang et al. 2022; Huang, Liu, and Tan 2020). In these studies, Huang and colleagues proposed the multicluster multigate mixture of experts model (M-MMOE). In the mixture of experts (MoE) model (Masoudnia and Ebrahimpour 2014), expert submodels are shared across all tasks and combined by a single gate. In the multigate mixture of experts (MMOE) (Ma et al. 2018) rather than a single gate across experts, a gating network is added for each task (see Figure 10B). In the M-MMOE, brain ROIs are first clustered using a novel algorithm, and then each cluster receives an MMOE which are themselves combined as experts. We chose to use the MMOE model in our comparison since it is well established and allowed us to explore if the simple addition of multiple experts and gates could improve the results of MTL by allowing the model to learn task relationships, rather than the M-MMOE structure which is unique to the Huang and colleagues studies and introduces much more complexity.

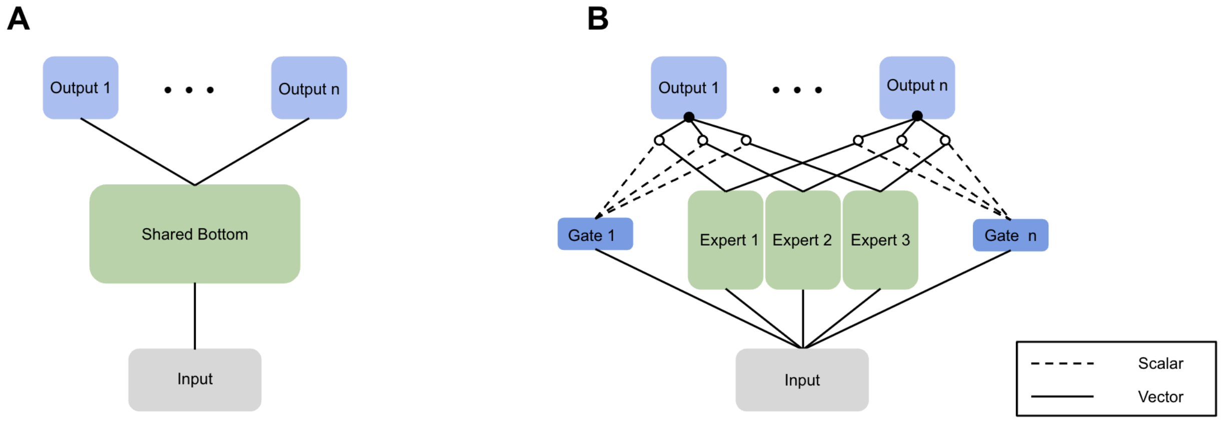

Figure 10 - A) Shared bottom model, B) MMOE.

We used the same encoder as in our MLPconn model as an expert, the same decoder as an output stack for each task, and followed the ratio of experts to tasks (2:1) used by Huang and colleagues. In detail, the input to the networks is a 1×2080 vector consisting of the upper triangular values of the symmetric connectome matrix, which is passed through each expert (two hidden layers with 256 and 64 units) as well as to the gating network (with N experts units), then the output from each expert is reweighted by the gate and summed, and passes finally to a task-specific output layer of 2 units for binary classification. Batch normalisation (Ioffe and Szegedy 2015) is applied after each layer. Training was implemented as described in the methods. First, we applied the MMOE across the full sample of conditions (22 experts, 11 tasks),

next to eliminate the complication of the small CNV datasets we applied the MLPconn and MMOE (8 experts, 4 tasks) models across only the psychiatric conditions in the dataset. Finally, we implemented the task relationship experiment with the MMOE model.

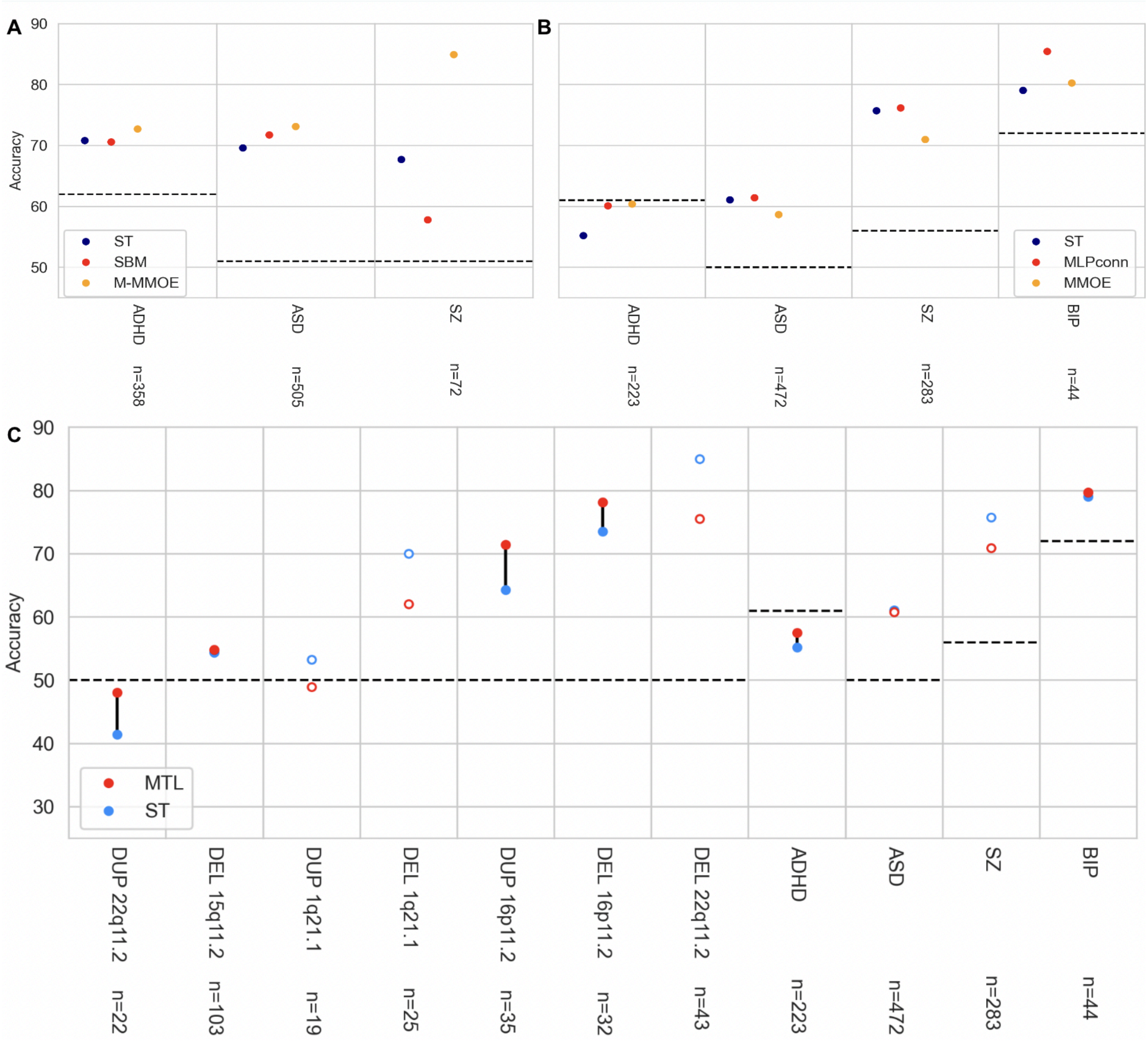

Figure 11 - A) Results reported in Huang et al. 2022 ST: Single Task, SBM: Shared Bottom Model, M-MMOE: variant of Multigate Mixture of Experts implemented by Huang and colleagues. Chance level indicated by a dashed line. B) Results of MMOE (8 experts) vs MLPconn across the psychiatric conditions C) Results of MMOE (22 experts) vs single task over the full set of conditions.

Using only the psychiatric conditions in the dataset, the MLPconn model improved prediction for all 4 conditions, whereas the MMOE helped accuracy for only 2 out of the 4 (see Figure 11B). This is contrary to the findings of Huang and colleagues, who reported marginal gains using their M-MMOE while the shared bottom model decreased accuracy (see Figure 11A). When we applied the MMOE across the full sample of conditions, we found that it performed slightly better than the MLPconn model on the full sample (accuracy improved for 6 out of 11 tasks vs. 4 out of 11) (see Figure 6). However, when examining task relationships using the MMOE (see

Figure 12) we saw a similar overall behaviour to the MLPconn model. Many pairs suffered a decrease in prediction accuracy when trained together, and the correlation with the results of the MLPconn model was high (r = 0.52).

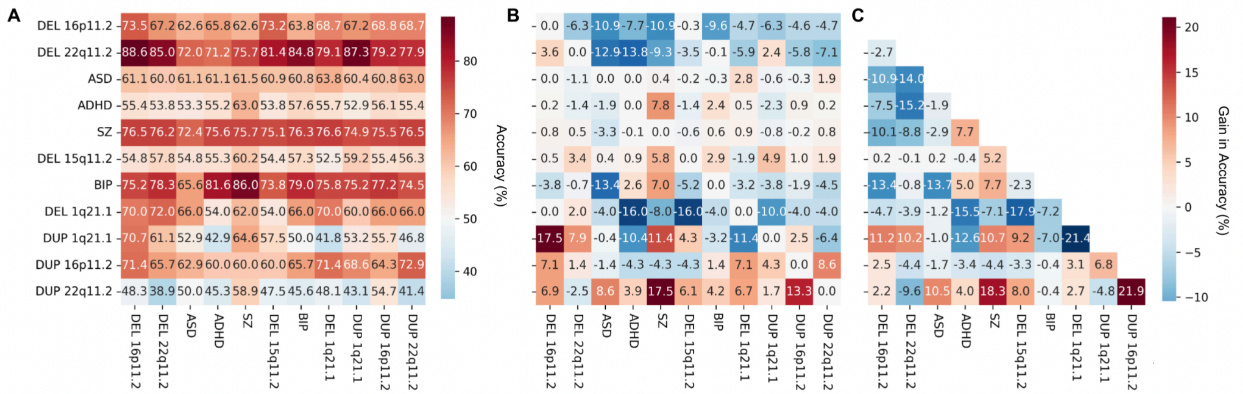

Figure 12 - In each matrix, the i,jth entry in the matrix is accuracy of condition in row i trained with condition in column j using the MLPconn model to perform automatic diagnosis. A: the matrix shows the raw accuracy achieved for each pair, the second matrix B represents the difference in accuracy from the single-task baseline, and the third C shows the overall gain relative to baseline for a pair ($B + B^T$).

A.4 - Distribution of Scores Across Folds of Cross-Validation

Here we present the distribution across the 5 folds of cross-validation of our results from sections 3.1.3, 3.1.4 and 3.2, rather than the average score, in order to give a better sense of the spread of MTL vs ST scores. We observed substantial variations in accuracy across folds, which was expected given the small sample size in each fold (less than 20 individuals, and as low as 6).

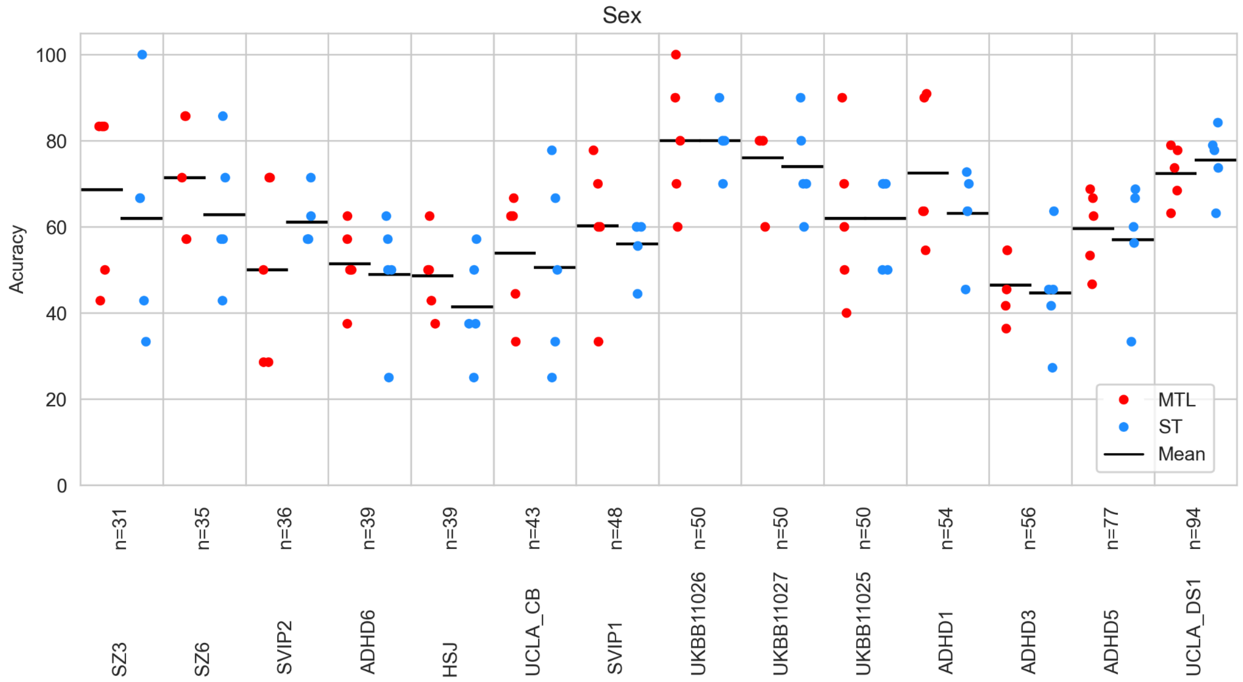

Figure 13 - Distribution of accuracy of sex prediction across 5-folds of k-fold cross-validation using single (ST) vs. multi-task learning (MTL) in a varied collection of sites. The x axis represents different data collection sites included as prediction tasks. Sites are ranked by sample size, with the largest to the right. The y axis shows the accuracy of prediction. For each task, the red points show prediction using the MLPconn architecture in MTL, and the blue points show prediction using the MLPconn architecture in ST.

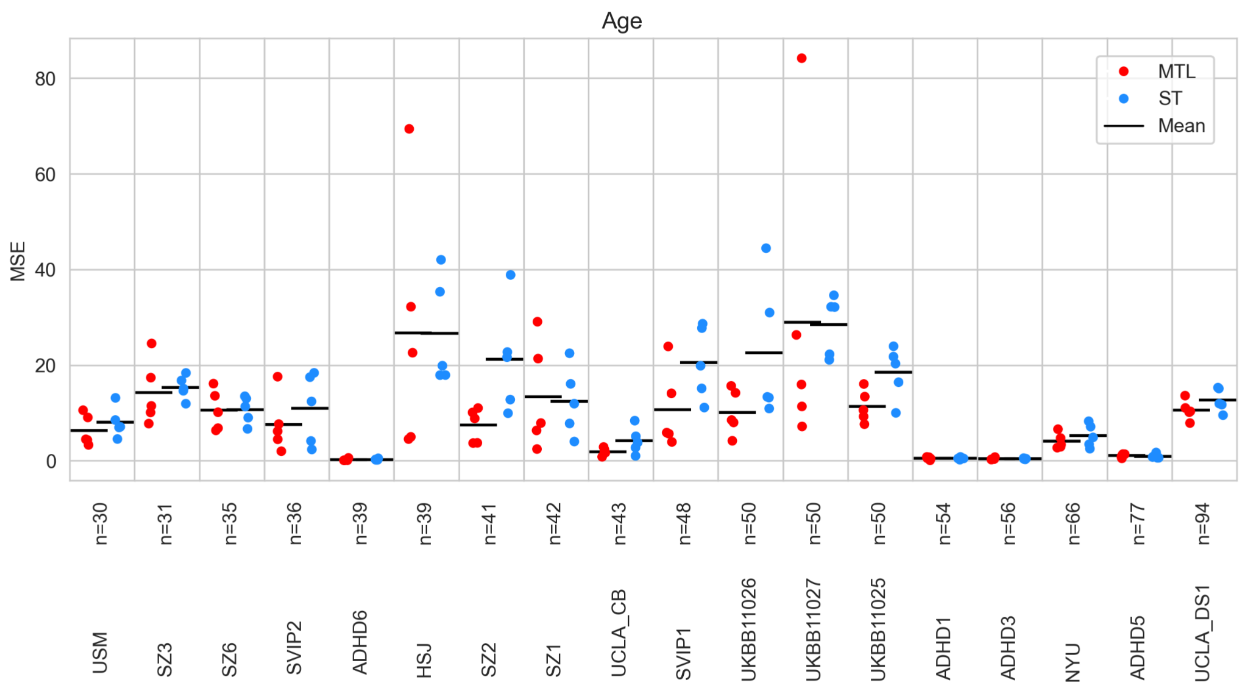

Figure 14 - Distribution of Mean Squared Error (MSE) of age prediction across 5-folds of k-fold cross-validation using single (ST) vs. multi-task learning (MTL) in a varied collection of sites. The x axis represents different data collection sites included as prediction tasks. Sites are ranked by sample size, with the largest to the right. The y axis shows the prediction error. For each task, the red points show prediction using the MLPconn_reg architecture in MTL, and the blue points show prediction using the MLPconn_reg architecture in ST.

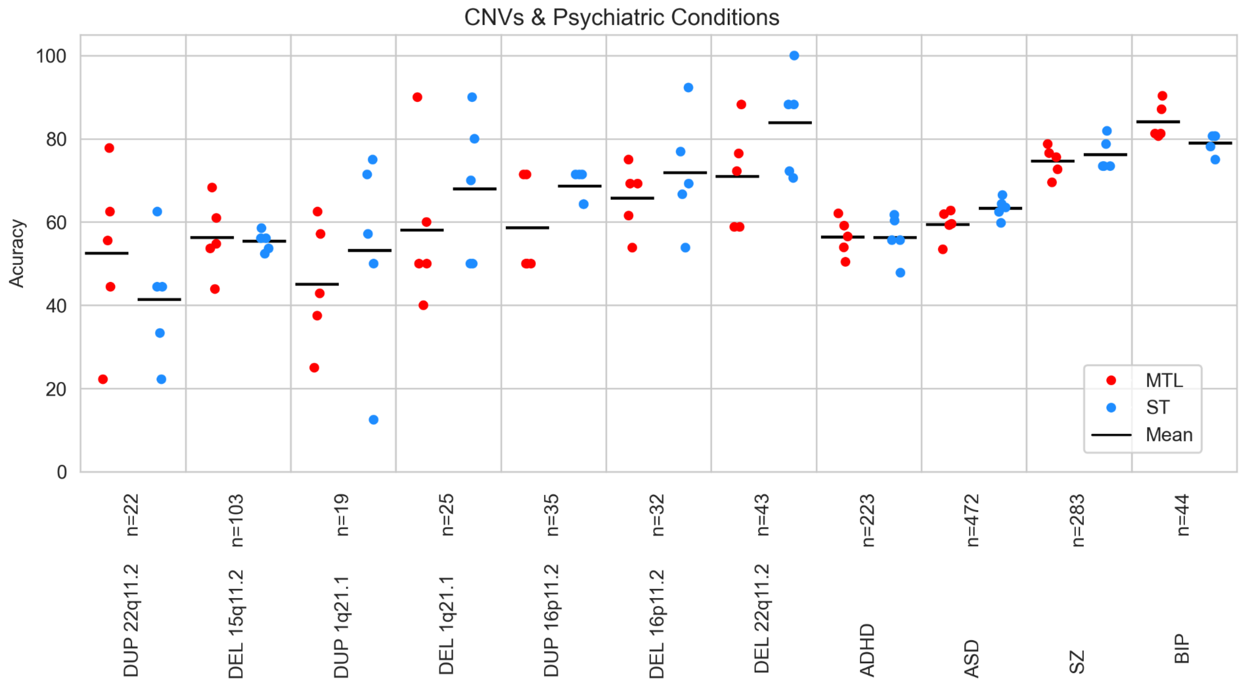

Figure 15 - Distribution of accuracy of automated diagnosis across 5-folds of k-fold cross-validation using single (ST) vs multi-task learning (MTL). The x axis represents different conditions included as prediction tasks. The y axis

shows the accuracy of prediction. For each task, the red points show prediction using the MLPconn architecture in MTL and the blue points show prediction using the MLPconn architecture in ST.

A.5 - AUC & F1 Score

Here we present the results of our classification prediction experiments, scored in sections 3.1.3 and 3.2 using prediction accuracy, using the Area Under the Receiver Operating Characteristic (AUC) (Nahm 2022) and F1-scores (Taha and Hanbury 2015) to provide a more comprehensive view of the model's performance. This is particularly relevant for the sex prediction study (section 3.1.3) in which the datasets have class imbalances (see Table 2).

These scores are derived from precision, recall (also called sensitivity), and specificity. Precision is defined as the number of true positives (subjects predicted as class 1 that are class 1) divided by the number of true positives plus false positives (subjects predicted as class 1 that are class 0). Recall is the number of true positives divided by the number of true positives plus false negatives (subjects predicted as class 0 that are class 1). Specificity is defined as the number of true negatives (subjects predicted as class 0 that are class 0) divided by the number of true negatives plus false positives. The F1-score is defined as the harmonic mean of precision and recall. In general classifiers output a continuous value which is turned into a binary prediction by comparing it to a threshold. The Receiver Operating Characteristic (ROC) curve plots recall vs. 1 - specificity at different thresholds. The AUC measures the overall performance of classification models using the area under the ROC curve. An AUC of 1 implies a perfect classifier and an AUC of 0.5 implies a random classifier. The qualitative conclusions of our experiments matched between AUC and accuracy scores, while F1 scores were more difficult to interpret.

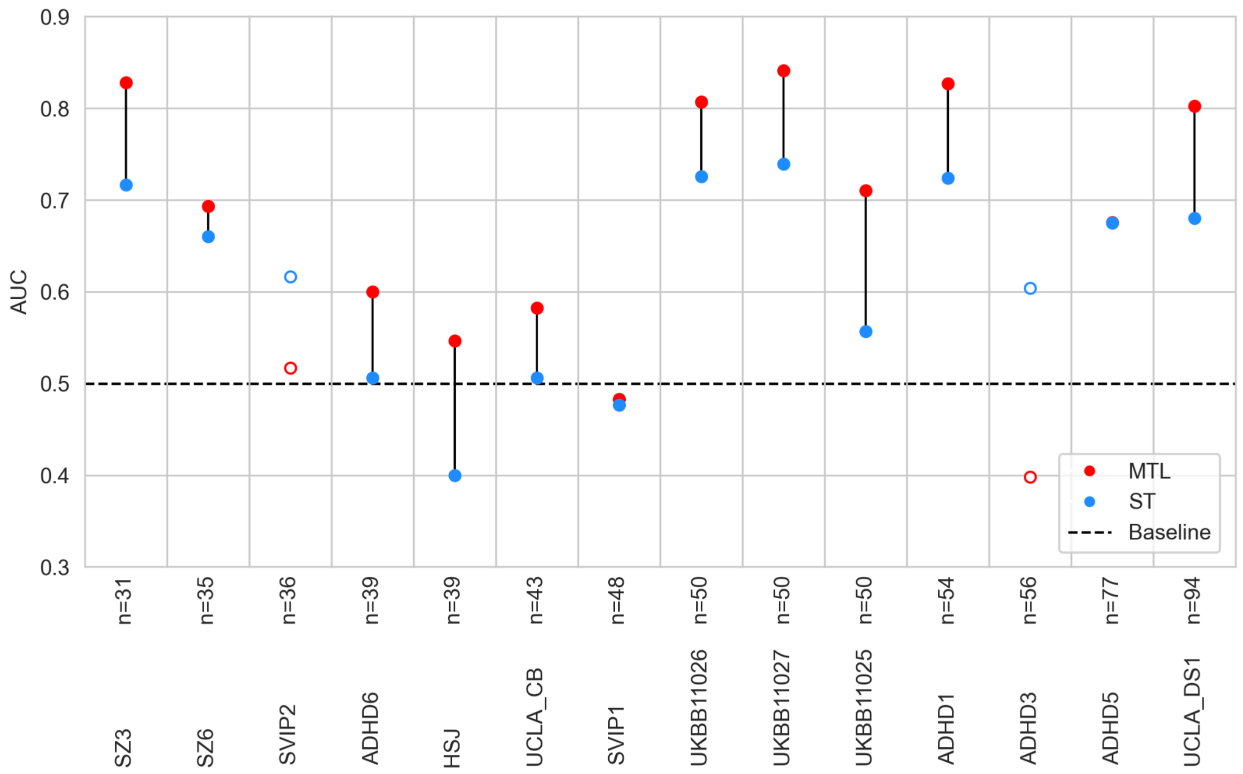

Figure 16 - Area Under the Receiver Operating Characteristic curve (AUC) of sex prediction using single (ST) vs. multi-task learning (MTL) in a varied collection of sites. The x axis represents different data collection sites included as prediction tasks. Sites are ranked by sample size, with the largest to the right. The y axis shows the AUC, chance level of prediction is indicated by a black dashed line. For each task, the red point shows prediction using the MLPconn architecture in MTL, and the blue point shows prediction on the task trained independently using the MLPconn architecture. Where the red point appears missing, the accuracy values for the two models are so close that the points are overlapped. If the MTL prediction outperformed the ST, points were filled and connected by a line, and otherwise they were not.

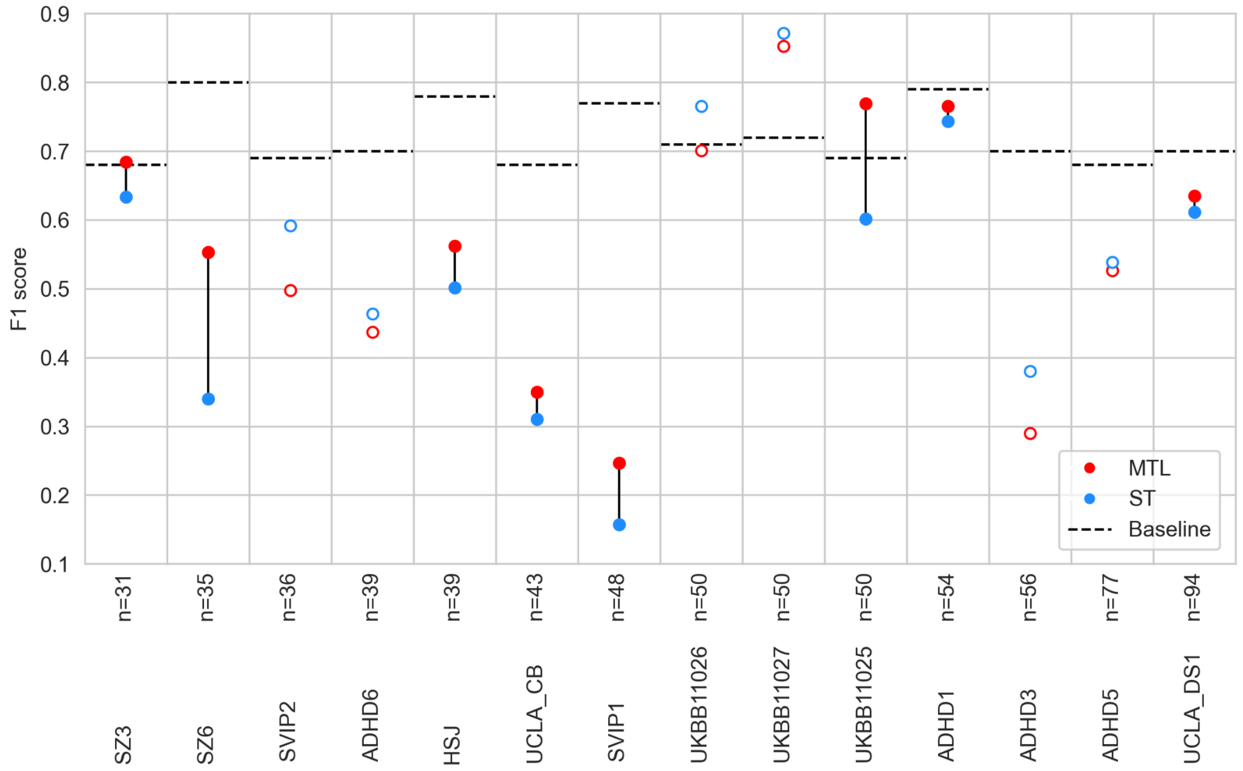

Figure 17 - F1 score of sex prediction using single (ST) vs. multi-task learning (MTL) in a varied collection of sites. The x axis represents different data collection sites included as prediction tasks. Sites are ranked by sample size, with the largest to the right. The y axis shows the F1 score of prediction, chance level of prediction is indicated by a black dashed line. For each task, the red point shows prediction using the MLPconn architecture in MTL, and the blue point shows prediction on the task trained independently using the MLPconn architecture. Where the red point appears missing, the accuracy values for the two models are so close that the points are overlapped. If the MTL

prediction outperformed the ST, points were filled and connected by a line, and otherwise they were not.

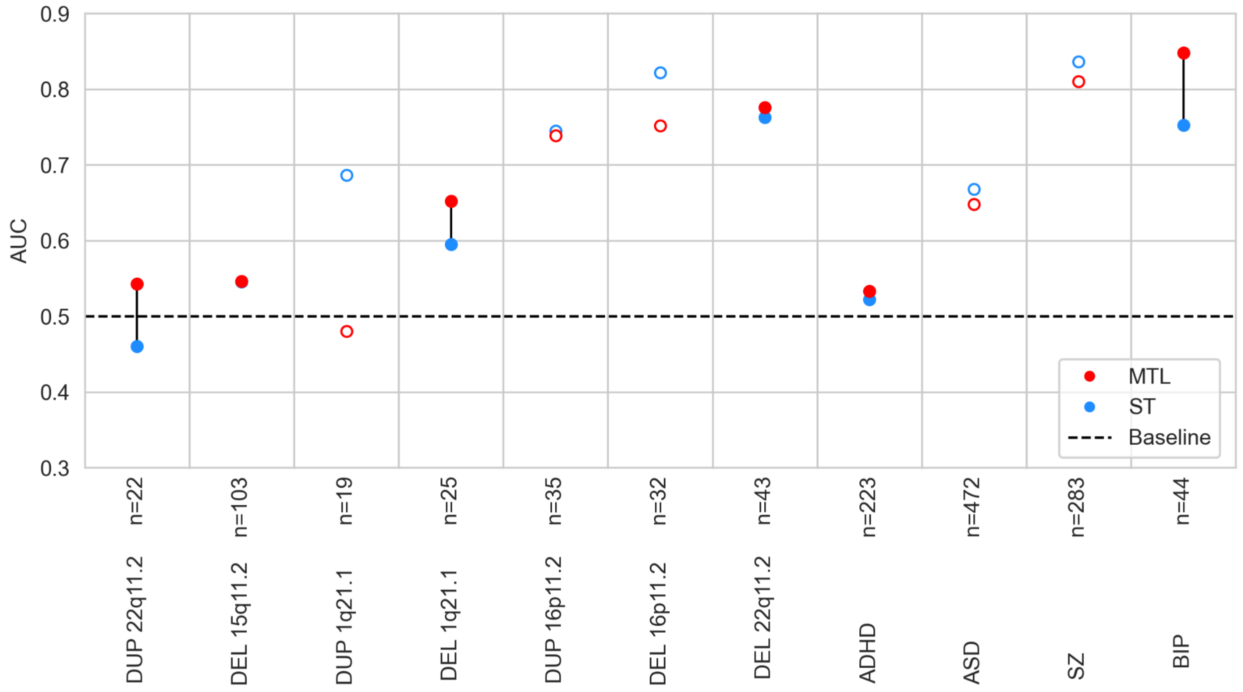

Figure 18 - Area Under the Receiver Operating Characteristic curve (AUC) of automated diagnosis using single (ST) vs multi-task learning (MTL). For each task, the red point shows prediction using the MLPconn architecture in MTL and the blue point shows prediction on the task trained independently using the MLPconn architecture. Where either the blue point appears missing, the accuracy values for the two models are so close that the points are overlapped. If the MTL prediction outperformed the ST, points are filled and connected by a line, and otherwise they are not. The x axis represents different conditions included as prediction tasks. The y axis shows the AUC of prediction, chance level of prediction is indicated by a black dashed line.

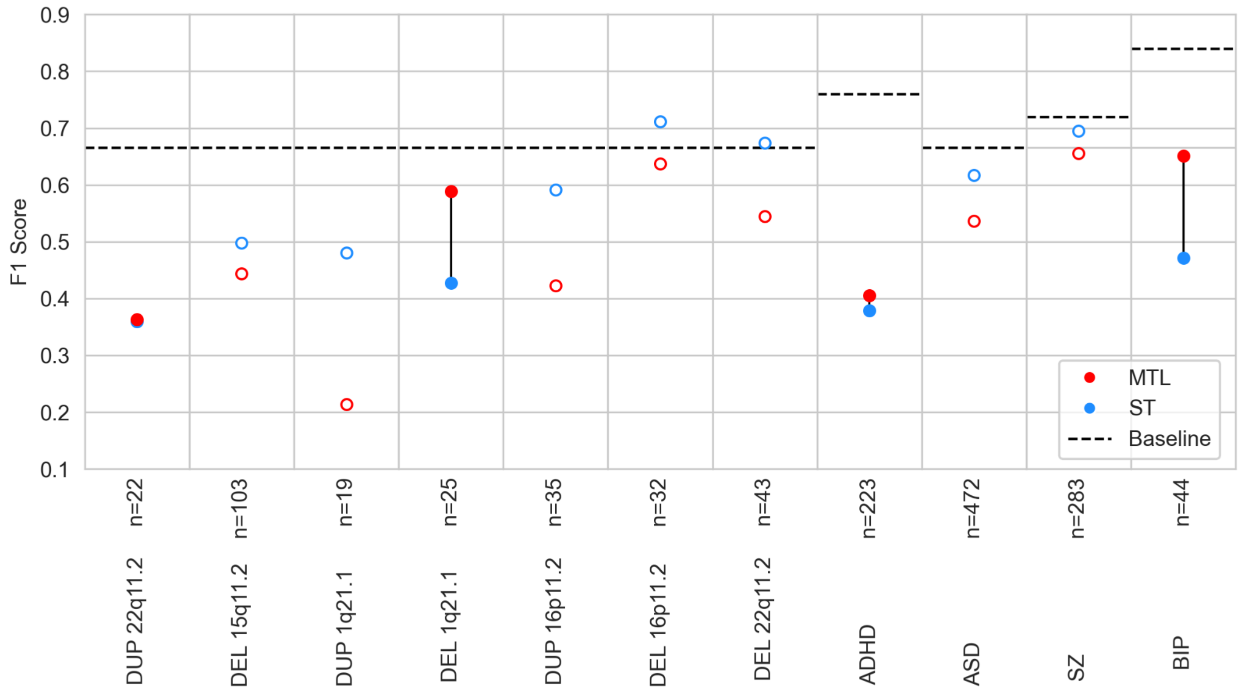

Figure 19 - F1 score of automated diagnosis using single (ST) vs multi-task learning (MTL). For each task, the red point shows prediction using the MLPconn architecture in MTL and the blue point shows prediction on the task trained independently using the MLPconn architecture. Where either the blue point appears missing, the accuracy values for the two models are so close that the points are overlapped. If the MTL prediction outperformed the ST, points are filled and connected by a line, and otherwise they are not. The x axis represents different conditions included as prediction tasks. The y axis shows the F1 score of prediction, chance level of prediction is indicated by a black dashed line.

A.6 - Effect Sizes as a Measure of Task Difficulty

Here we aimed to measure how difficult each prediction task is in order to contextualise the performance of MTL in different settings: predicting age or sex across sites of data collection, and performing automatic diagnosis across CNVs and psychiatric conditions.

Traditional fMRI research often approaches group comparisons using traditional regression models applied independently on each feature (brain connection), a technique called connectome-wide association study (CWAS). In this context, the most classic measure of "task difficulty" is so-called Cohen's d estimate, which is the difference in average between two groups, relative to the standard deviation of the feature within-group. We would like to emphasise that there is no theoretical reason for CWAS effect sizes to match accuracy with ML tools, as ML tools are a multivariate measure of effect size (akin to a statistical omnibus tests) rather than mass univariate like CWAS. In practice these two types of effect sizes do not necessarily align (Bzdok and Ioannidis 2019; Shmueli 2010; Lo et al. 2015). However, CWAS effect sizes are a common metric and provide intuitive guidance for interpretations.

Specifically, we implemented 13 CWAS using sex as a contrast for each site included in the sex prediction study, 18 CWAS using age groups as a contrast (younger half of subjects vs. older half) for each site included in age prediction study, and 11 CWAS for the following conditions: 7 CNVs and 4 psychiatric conditions. For the CWAS on conditions, control subjects refers to individuals without a CNV for analysis investigating the effect of CNVs, and individuals without a diagnosis in analyses investigating effects of psychiatric conditions. In order to have the best possible statistical power, we pooled all the control subjects we had access to (n = 31425, 16590 female subjects, age mean 62.31 and standard deviation 11.47, framewise displacement mean 0.18 and standard deviation 0.05, from a total of 53 sites of data collection). The results on CNVs and psychiatric conditions presented here were published in two studies: (Moreau et al. 2023) and (Moreau et al. 2022).

For each CWAS, we applied linear regression independently for each of the 2080 values of the connectome: the FC values were first z-scored based on the variance of the relevant control subjects, so the regression estimates can also be interpreted as z-scores, and then used as the dependent variable with the genetic or diagnostic status as the explanatory variable. For the CWAS on sex at each site, models were head motion, age and global signal. For the CWAS on age at each site, models were head motion, sex and global signal. For the CWAS on conditions, models were adjusted for sex, scanning site, head motion, age and global signal. Global signal was defined as the mean of the connectome, and was included in the analysis as it has been shown that global signal-adjusted FC profiles show stronger correlations with cognition (Li et al. 2019) and reduce confounding effects in multisite studies (Yan et al. 2013). FC profiles were defined as the 2080 beta values of 2080 connections from each CWAS. The significance of beta values corrected for multiple tests using the Benjamini-Hochberg false discovery rate (FDR)

correction (Benjamini and Hochberg 1995) at a threshold of $q < 0.05$. We defined effect size on connectivity as the mean of the top decile of the absolute value of the 2080 beta values in the FC profile (Moreau et al. 2023).

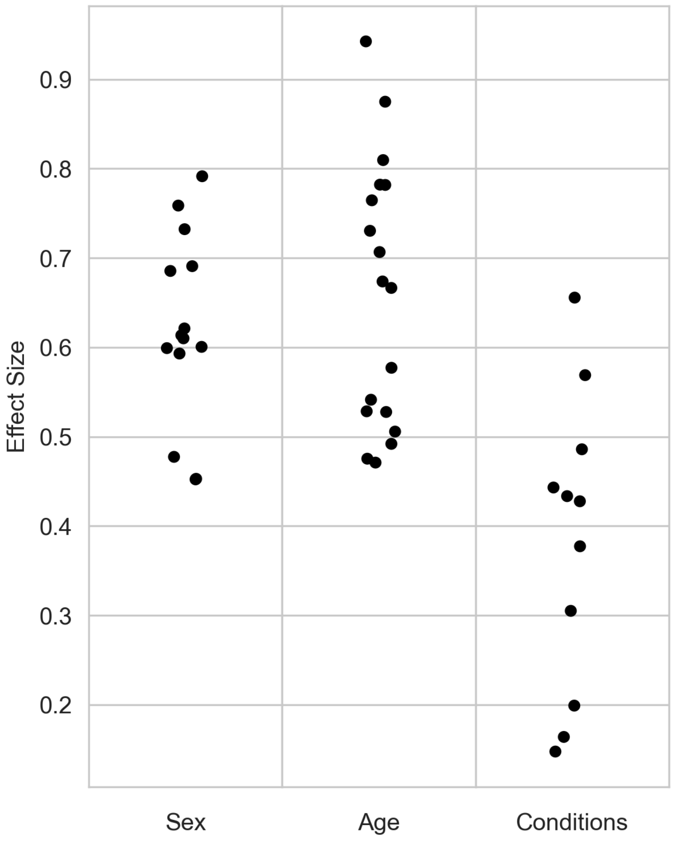

Figure 20 - Effect sizes on connectivity, defined as the mean of the top decile of the FC profile, for sex and age at each site of data collection and conditions (7 CNVs and 4 psychiatric conditions).

Effect sizes on connectivity for sex and age (at 14 and 18 scanning sites respectively) are much higher than for the 7 CNVs and 4 psychiatric conditions included in our dataset. This matches the observed behaviour of ML techniques in the case of predicting sex vs predicting conditions, as the accuracy of sex classification is higher in the full UK biobank sample (see section 3.1.1) than any of the automated diagnostic classifiers (see section 10.1). Predicting age is more difficult to directly compare with automatic diagnosis since it is a regression rather than a classification task. Overall, the higher effect sizes for sex and age relative to conditions makes them easier as prediction tasks in the ST setting and therefore more likely to benefit multi-task learning. Additionally, predicting sex or age across different scanning sites is intuitively better suited for MTL since the tasks have a common target and therefore clearly have shared information that can be exploited by a combined model. In the case of automatic diagnosis, while the conditions are related and have substantial shared information, they are all distinct conditions and therefore less easily combined by a single MTL model.

A.7 - Confound distributions by Site & Condition

Here we present plots of the distribution of the confounding variables for each dataset, first the single scanning site datasets (control subjects only) used for the age & sex prediction tasks (sections 3.1.3 and 3.1.4) followed by the multi-site datasets used to predict conditions in section 3.2 (matched number of cases & controls). These demonstrate the large dataset variability regarding confound distribution, which provides important context on the results from the previous studies, and is also important to interpret the ablation study (section 10.8) in which MTL prediction is repeated using all but one dataset in order to analyse the effect of each dataset on performance.

A.7.1 - Single Site Datasets

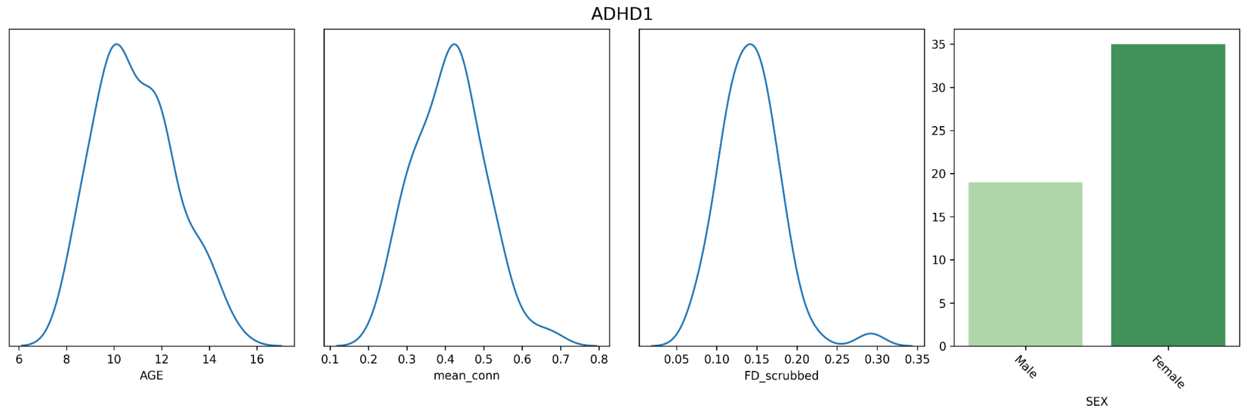

Figure 21 - Distribution of confounding variables (age, global signal (mean_conn), head motion (FD_scrubbed), and sex) among control subjects at the ADHD1 scanning site.

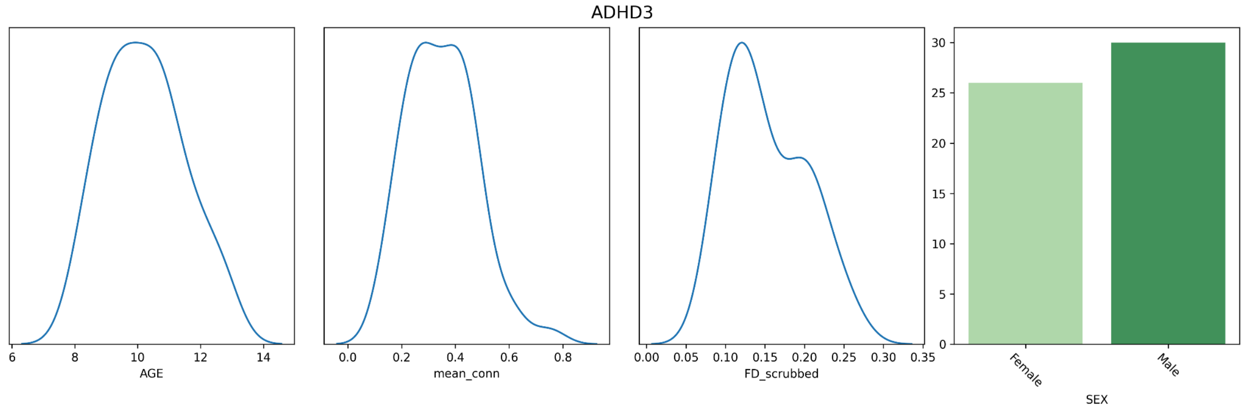

Figure 22 - Distribution of confounding variables (age, global signal (mean_conn), head motion (FD_scrubbed), and sex) among control subjects at the ADHD3 scanning site.

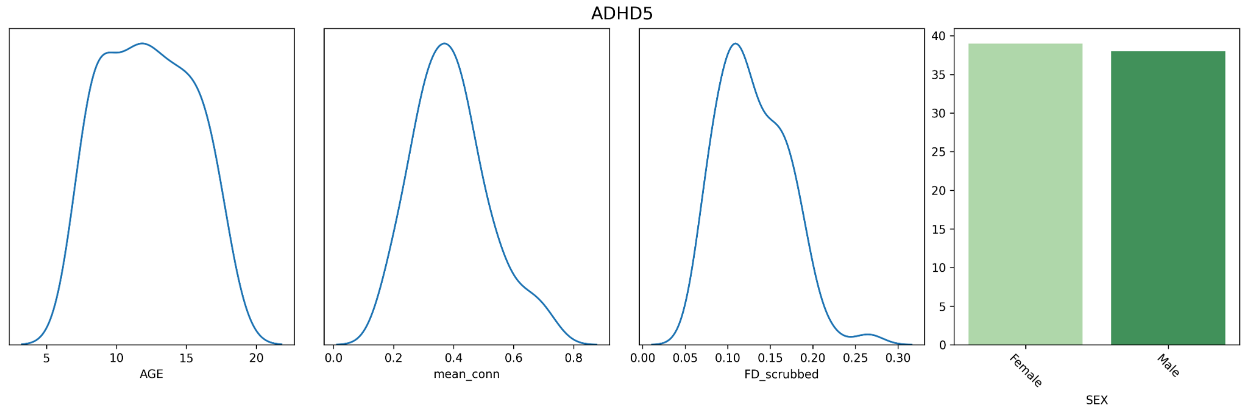

Figure 23 - Distribution of confounding variables (age, global signal (mean_conn), head motion (FD_scrubbed), and sex) among control subjects at the ADHD5 scanning site.

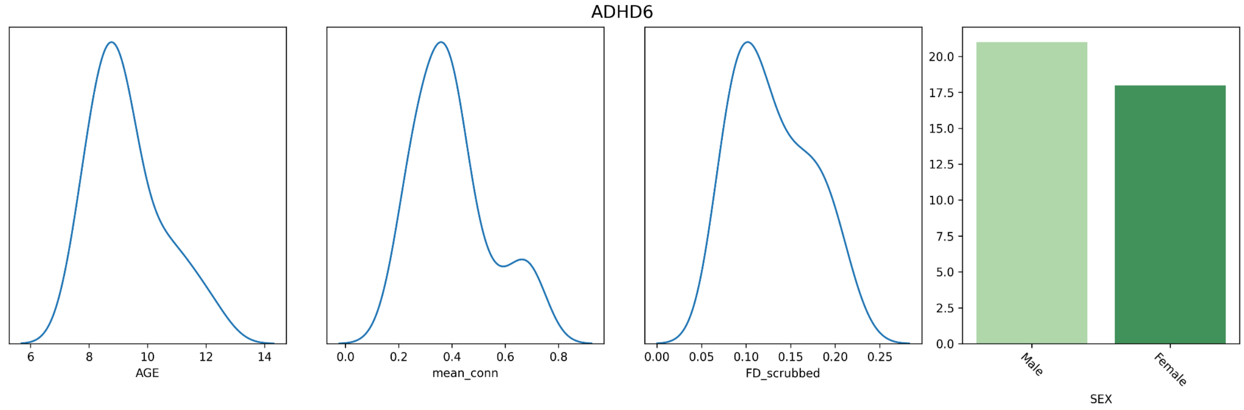

Figure 24 - Distribution of confounding variables (age, global signal (mean_conn), head motion (FD_scrubbed), and sex) among control subjects at the ADHD6 scanning site.

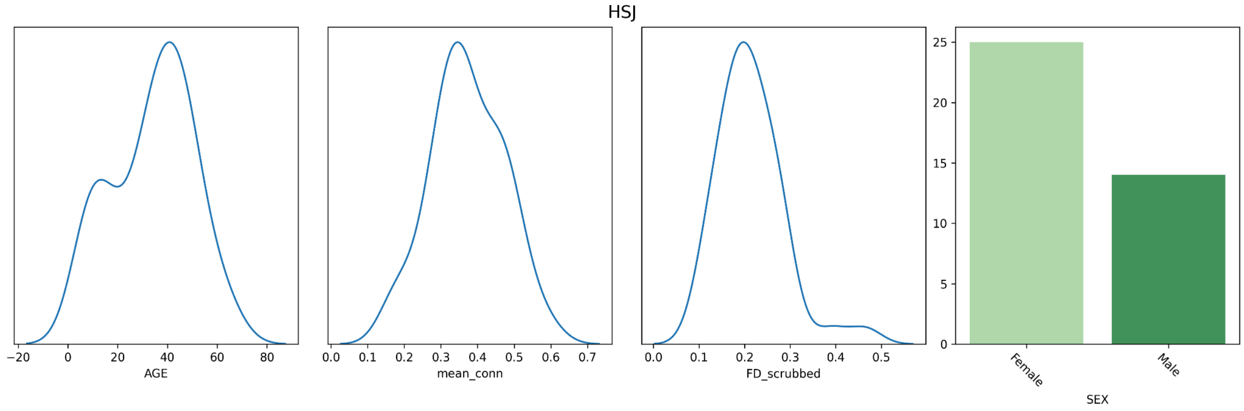

Figure 25 - Distribution of confounding variables (age, global signal (mean_conn), head motion (FD_scrubbed), and sex) among control subjects at the HSJ scanning site.

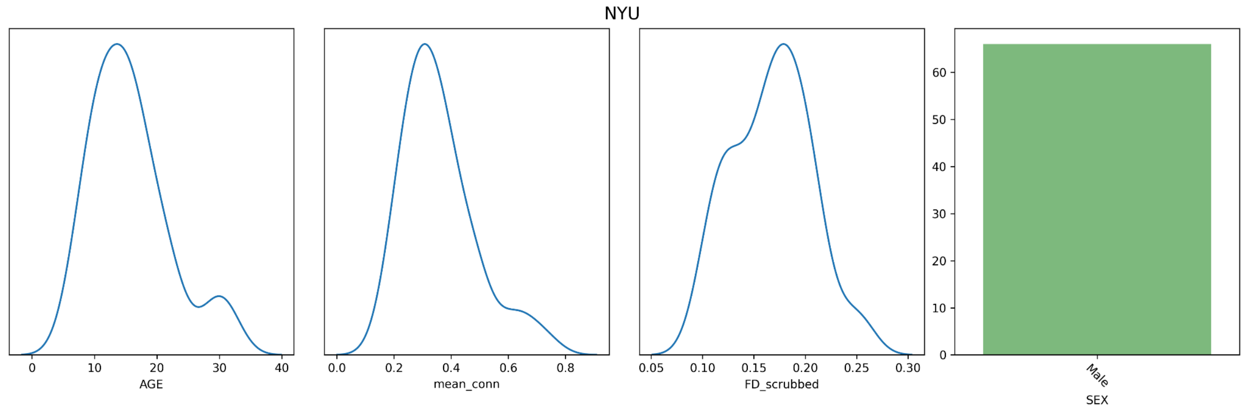

Figure 26 - Distribution of confounding variables (age, global signal (mean_conn), head motion (FD_scrubbed), and sex) among control subjects at the NYU scanning site.

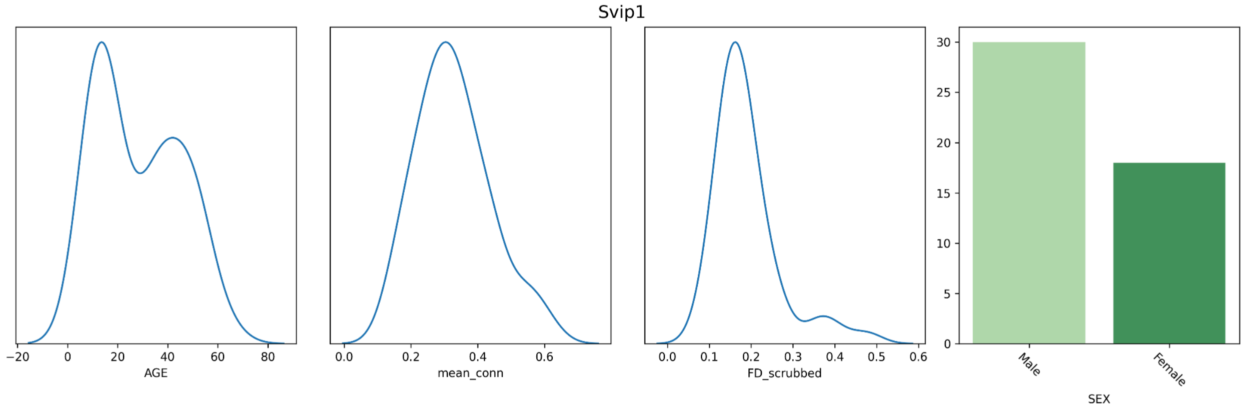

Figure 27 - Distribution of confounding variables (age, global signal (mean_conn), head motion (FD_scrubbed), and sex) among control subjects at the Svip1 scanning site.

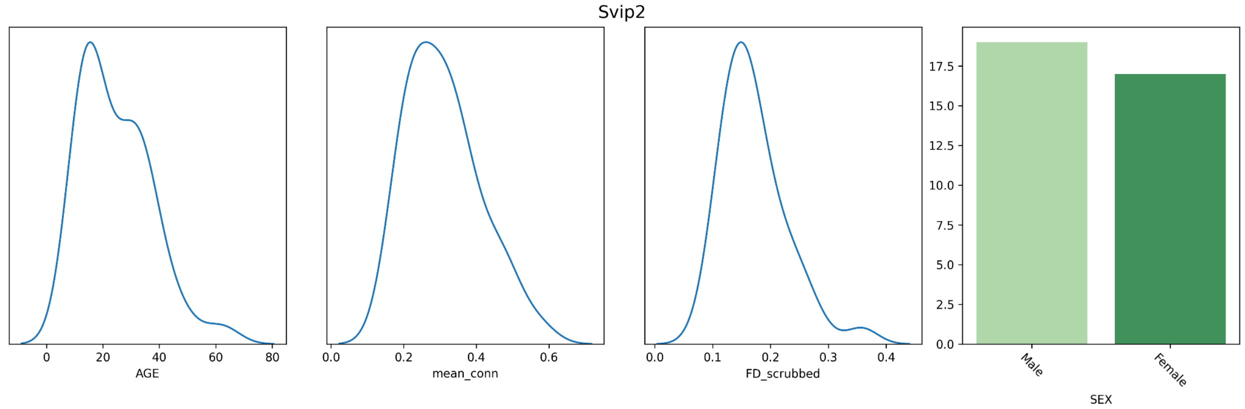

Figure 28 - Distribution of confounding variables (age, global signal (mean_conn), head motion (FD_scrubbed), and sex) among control subjects at the Svip2 scanning site.

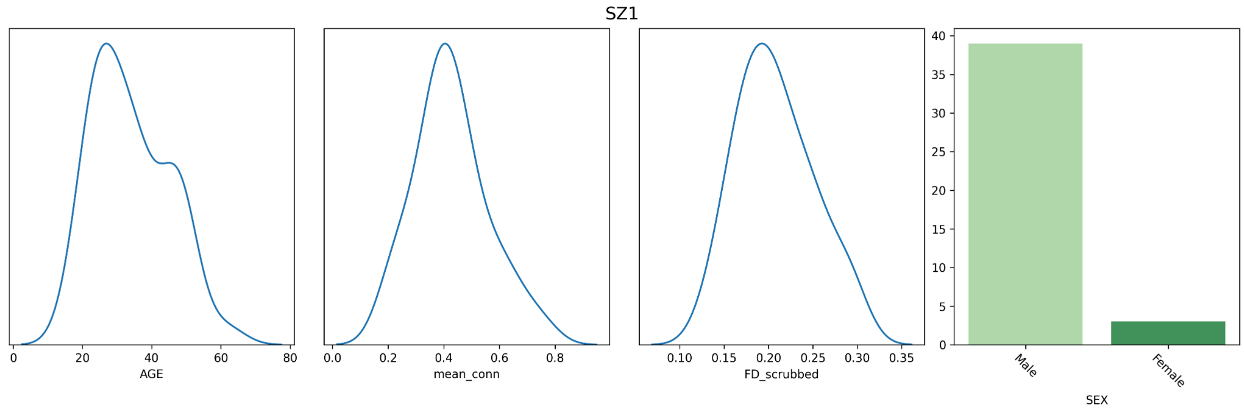

Figure 29 - Distribution of confounding variables (age, global signal (mean_conn), head motion (FD_scrubbed), and sex) among control subjects at the SZ1 scanning site.

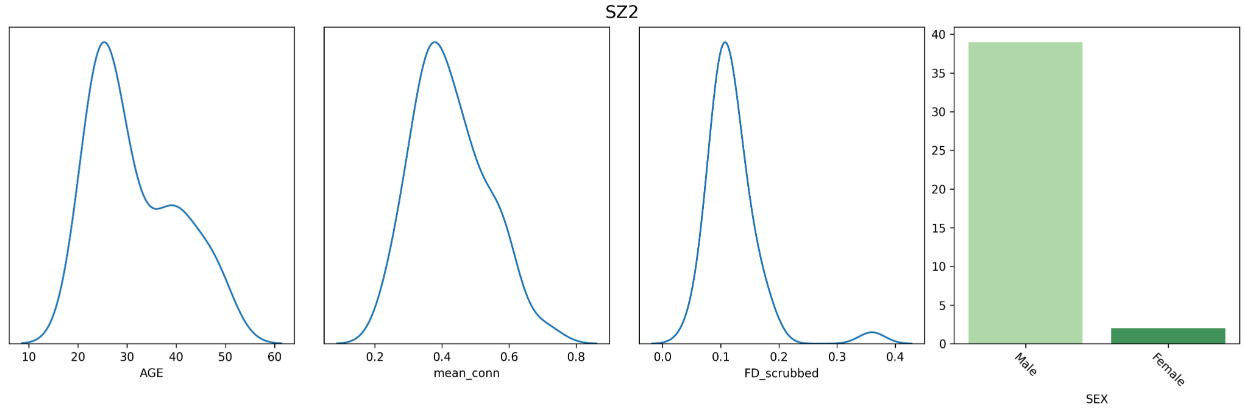

Figure 30 - Distribution of confounding variables (age, global signal (mean_conn), head motion (FD_scrubbed), and sex) among control subjects at the SZ2 scanning site.

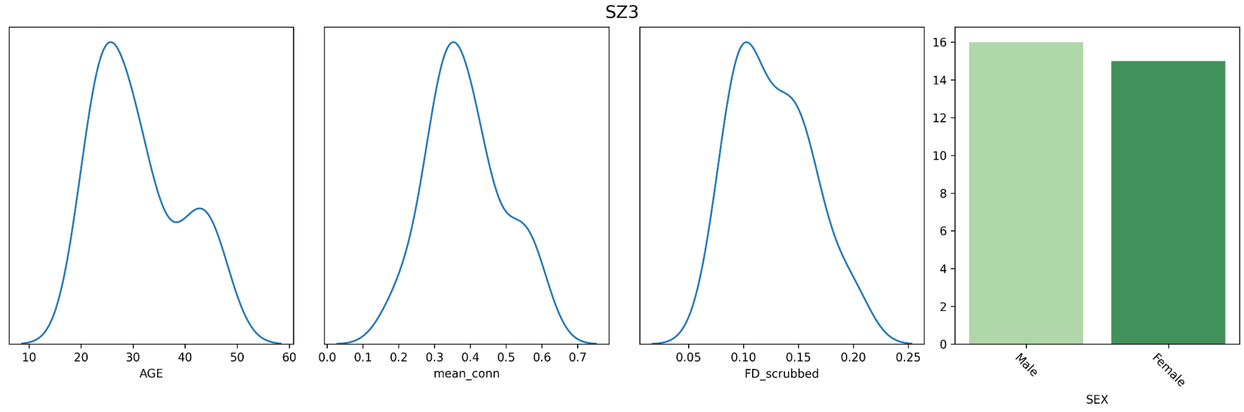

Figure 31 - Distribution of confounding variables (age, global signal (mean_conn), head motion (FD_scrubbed), and sex) among control subjects at the SZ3 scanning site.

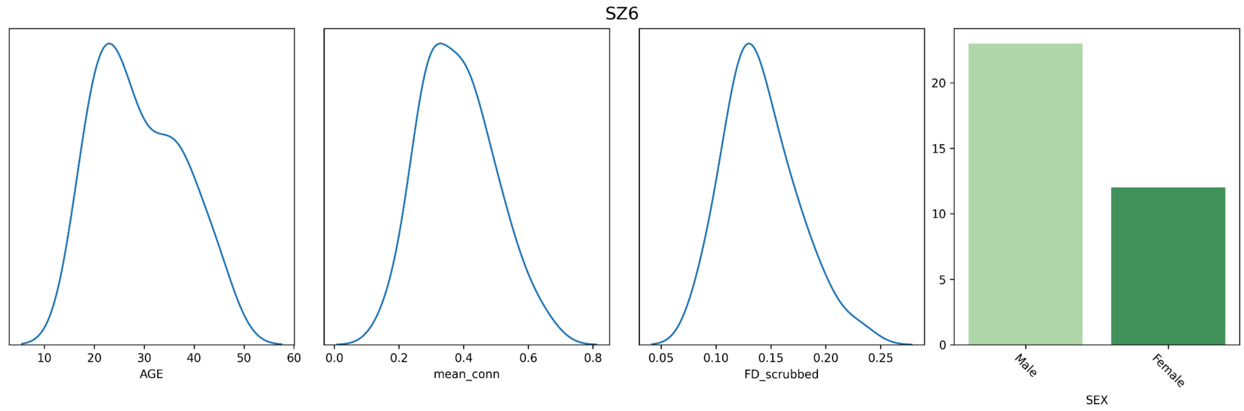

Figure 32 - Distribution of confounding variables (age, global signal (mean_conn), head motion (FD_scrubbed), and sex) among control subjects at the SZ6 scanning site.

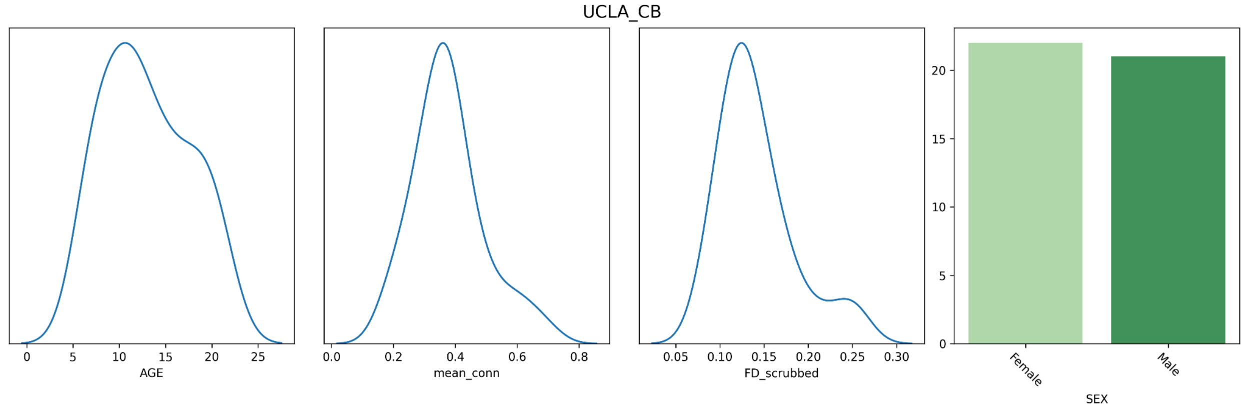

Figure 33 - Distribution of confounding variables (age, global signal (mean_conn), head motion (FD_scrubbed), and sex) among control subjects at the UCLA_CB scanning site.

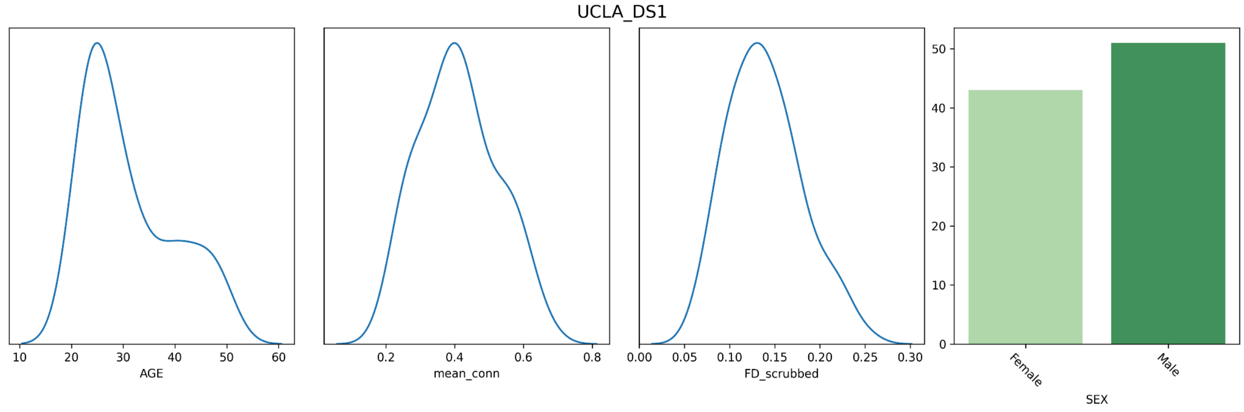

Figure 34 - Distribution of confounding variables (age, global signal (mean_conn), head motion (FD_scrubbed), and sex) among control subjects at the UCLA_DS1 scanning site.

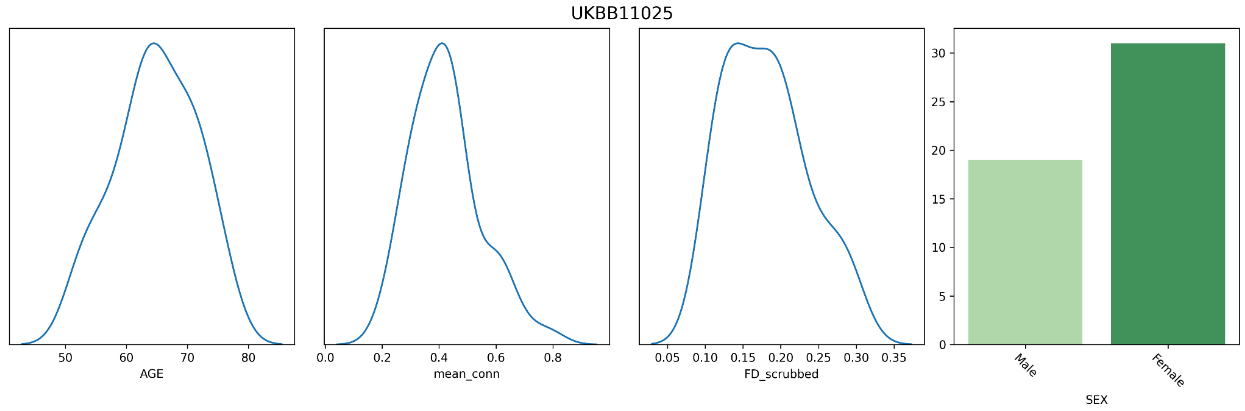

Figure 35 - Distribution of confounding variables (age, global signal (mean_conn), head motion (FD_scrubbed), and sex) among 50 subsampled control subjects at the UKBB11025 scanning site.

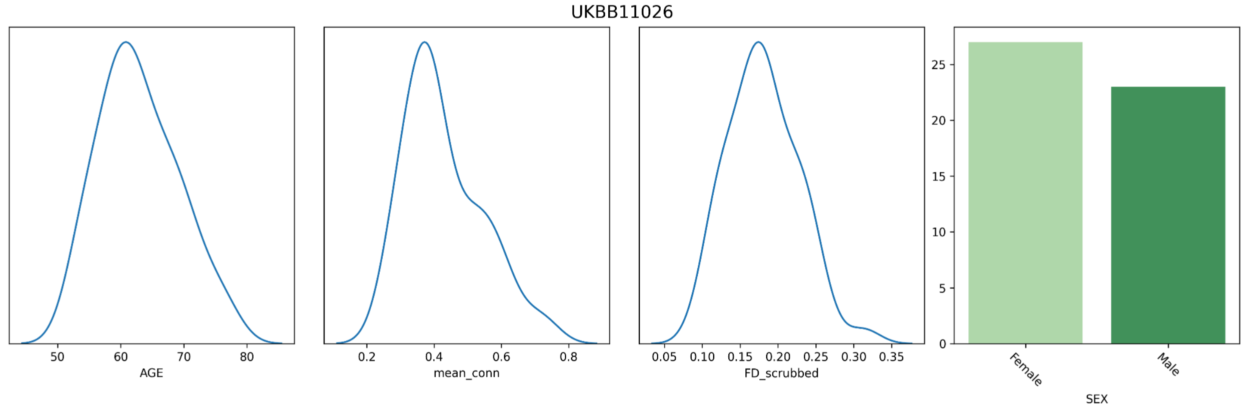

Figure 36 - Distribution of confounding variables (age, global signal (mean_conn), head motion (FD_scrubbed), and sex) among 50 subsampled control subjects at the UKBB11026 scanning site.

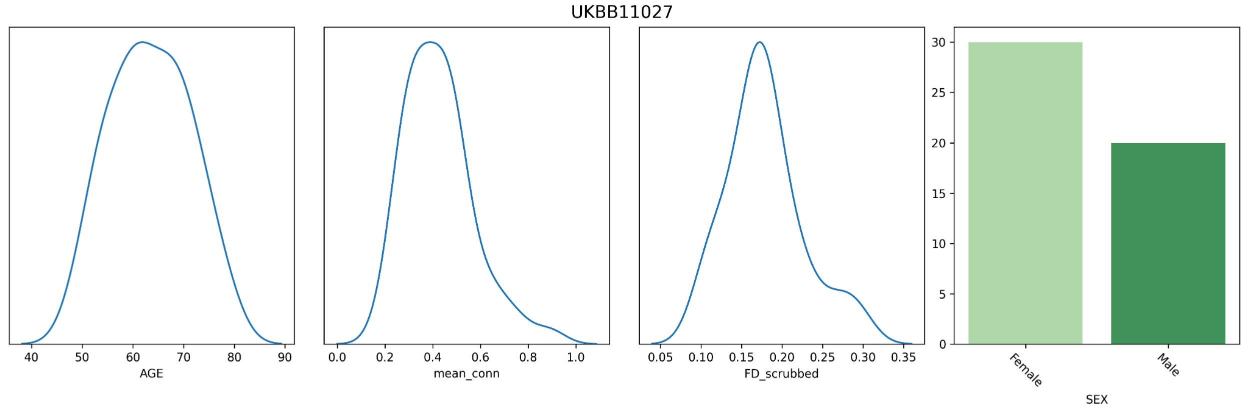

Figure 37 - Distribution of confounding variables (age, global signal (mean_conn), head motion (FD_scrubbed), and sex) among 50 subsampled control subjects at the UKBB11027 scanning site.

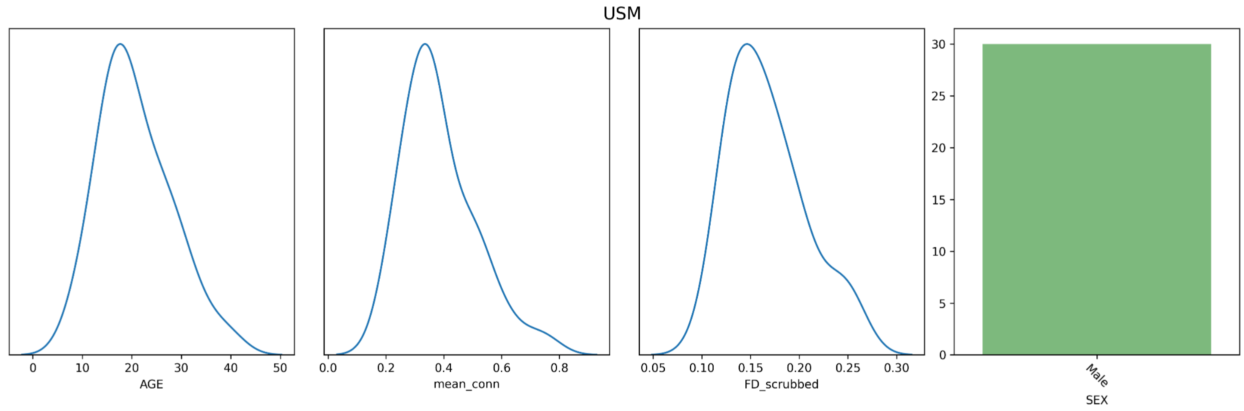

Figure 38 - Distribution of confounding variables (age, global signal (mean_conn), head motion (FD_scrubbed), and sex) among control subjects at the USM scanning site.

A.7.2 - CNV & Psychiatric Condition Datasets

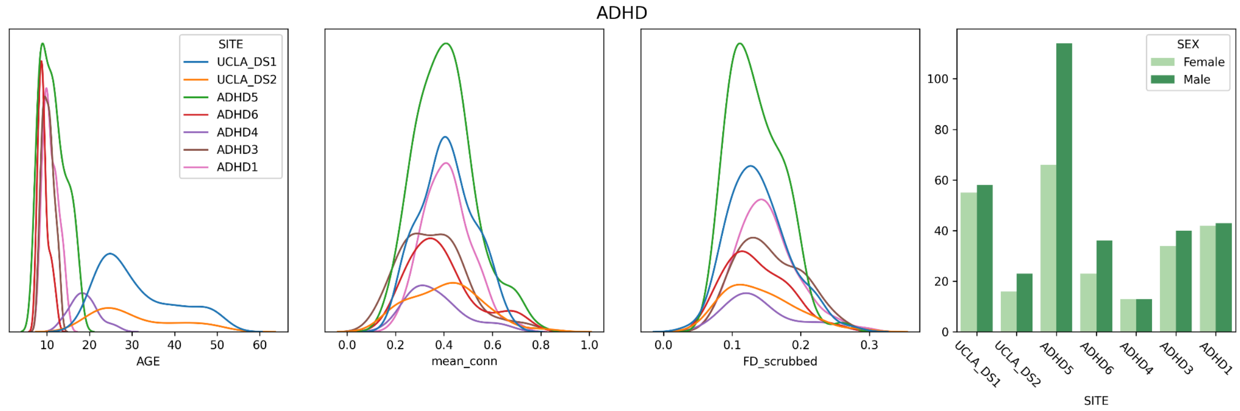

Figure 39 - Distribution of confounding variables (age, global signal (mean_conn), head motion (FD_scrubbed), and sex) by scanning site for the ADHD dataset.

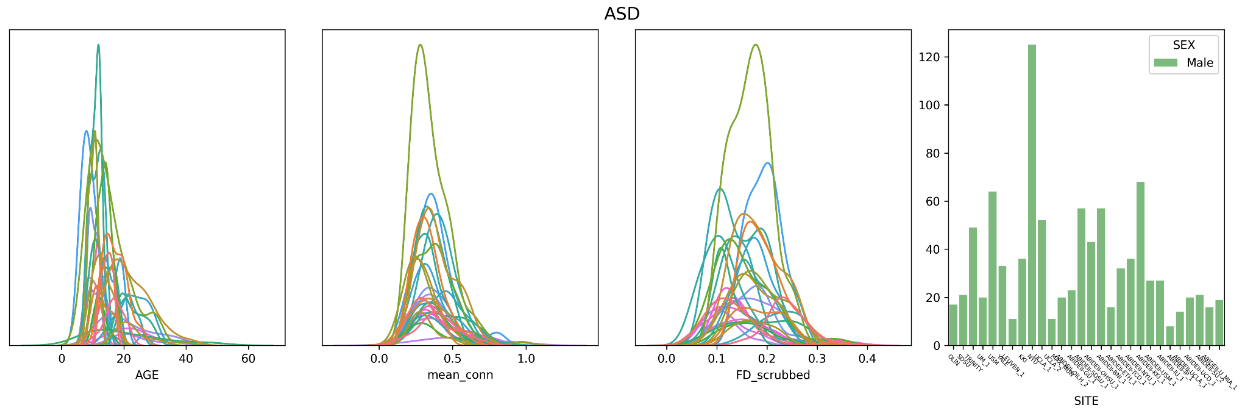

Figure 40 - Distribution of confounding variables (age, global signal (mean_conn), head motion (FD_scrubbed), and sex) by scanning site for the ASD dataset.

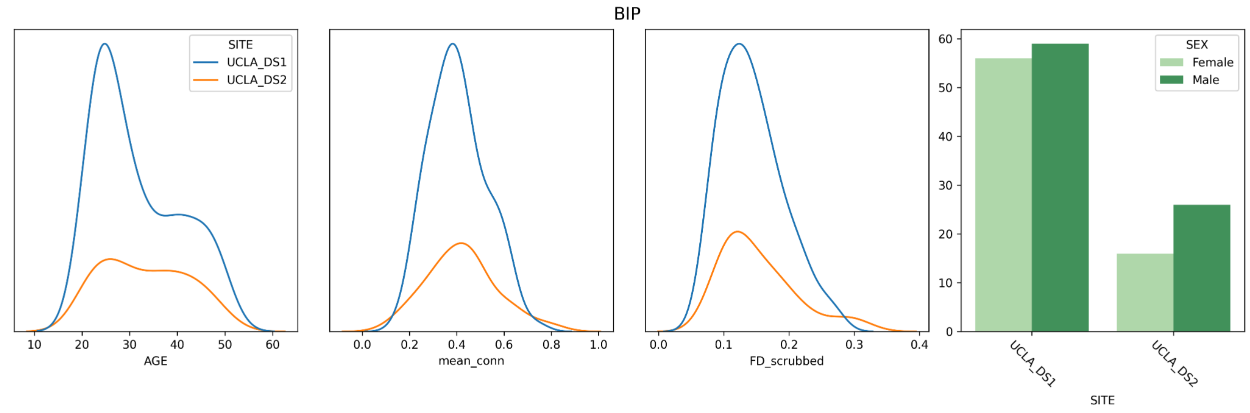

Figure 41 - Distribution of confounding variables (age, global signal (mean_conn), head motion (FD_scrubbed), and sex) by scanning site for the BIP dataset.

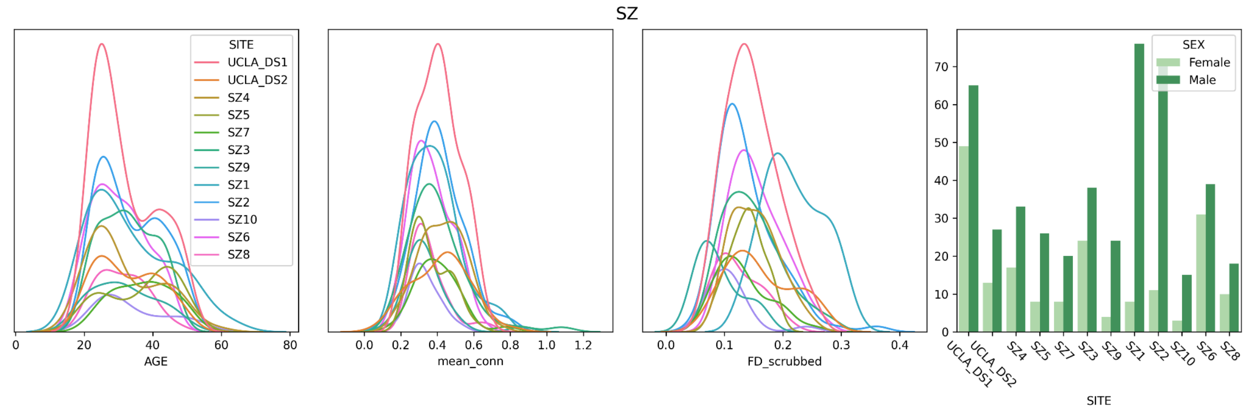

Figure 42 - Distribution of confounding variables (age, global signal (mean_conn), head motion (FD_scrubbed), and sex) by scanning site for the SZ dataset.

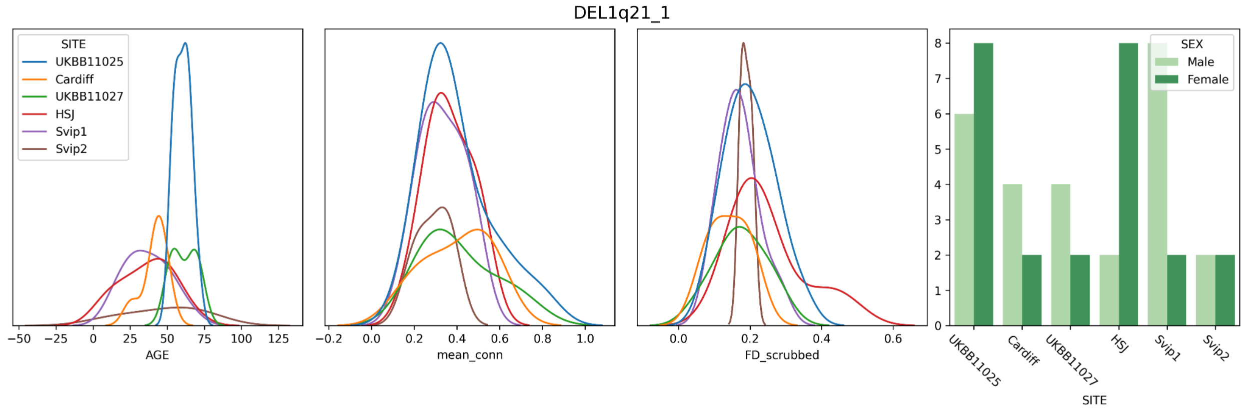

Figure 43 - Distribution of confounding variables (age, global signal (mean_conn), head motion (FD_scrubbed), and sex) by scanning site for the DEL 1q21.1 dataset.

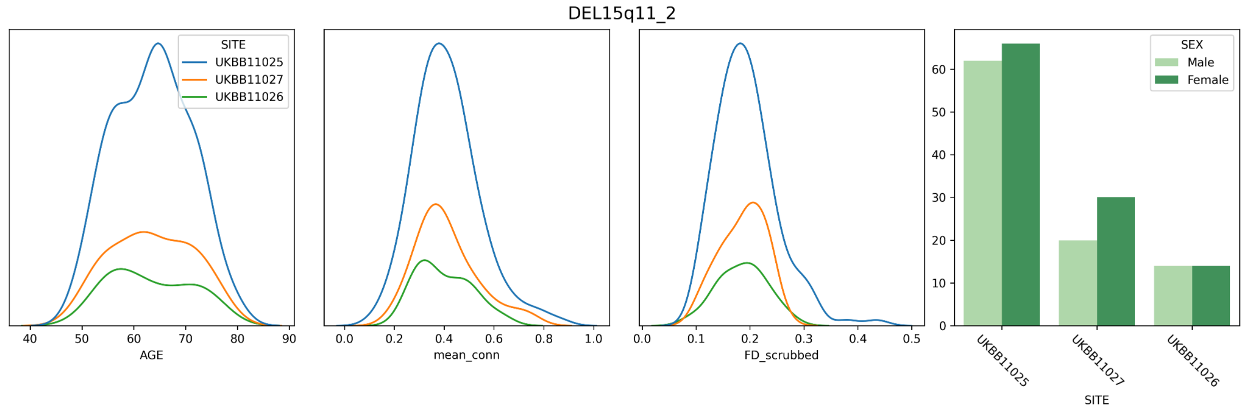

Figure 44 - Distribution of confounding variables (age, global signal (mean_conn), head motion (FD_scrubbed), and sex) by scanning site for the DEL 15q11.2 dataset.

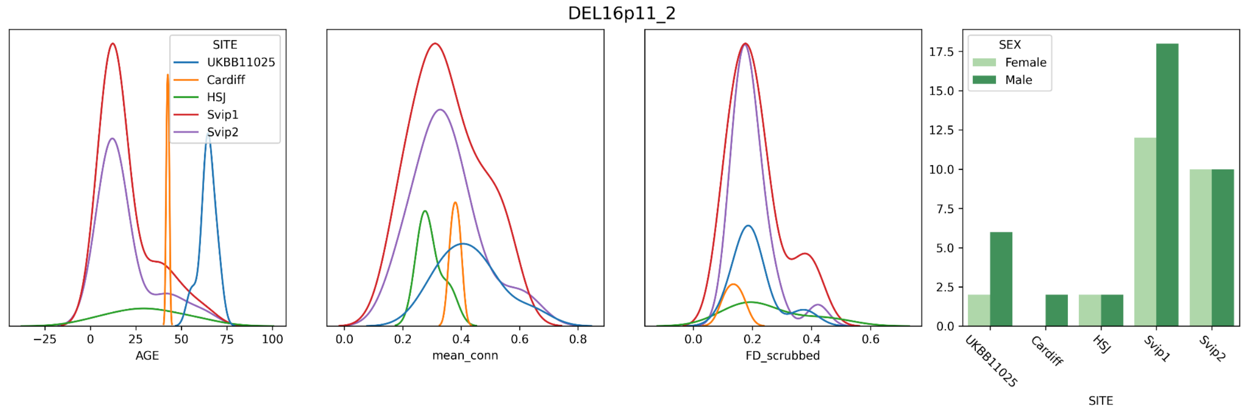

Figure 45 - Distribution of confounding variables (age, global signal (mean_conn), head motion (FD_scrubbed), and sex) by scanning site for the DEL 16p11.2 dataset.

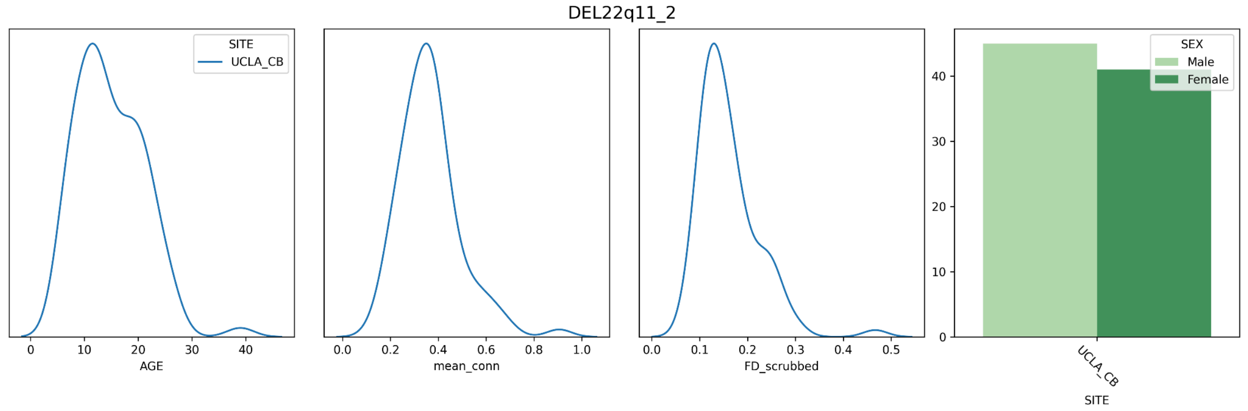

Figure 46 - Distribution of confounding variables (age, global signal (mean_conn), head motion (FD_scrubbed), and sex) by scanning site for the DEL 22q11.2 dataset.

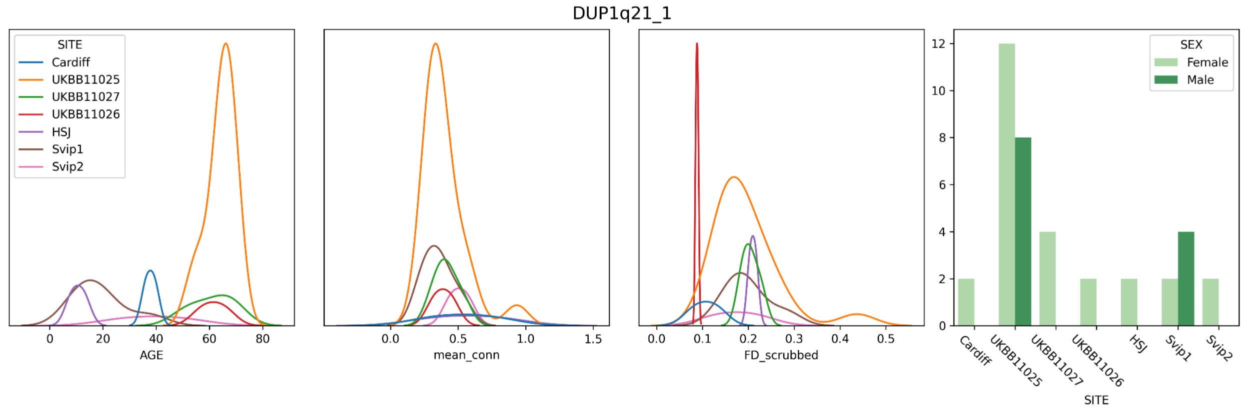

Figure 47 - Distribution of confounding variables (age, global signal (mean_conn), head motion (FD_scrubbed), and sex) by scanning site for the DUP 1q21.1 dataset.

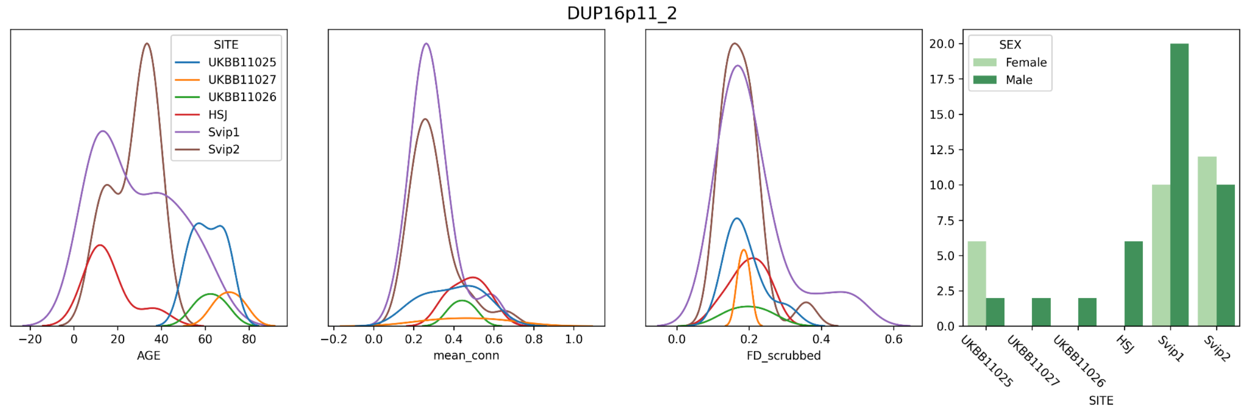

Figure 48 - Distribution of confounding variables (age, global signal (mean_conn), head motion (FD_scrubbed), and sex) by scanning site for the DUP 16p11.2 dataset.

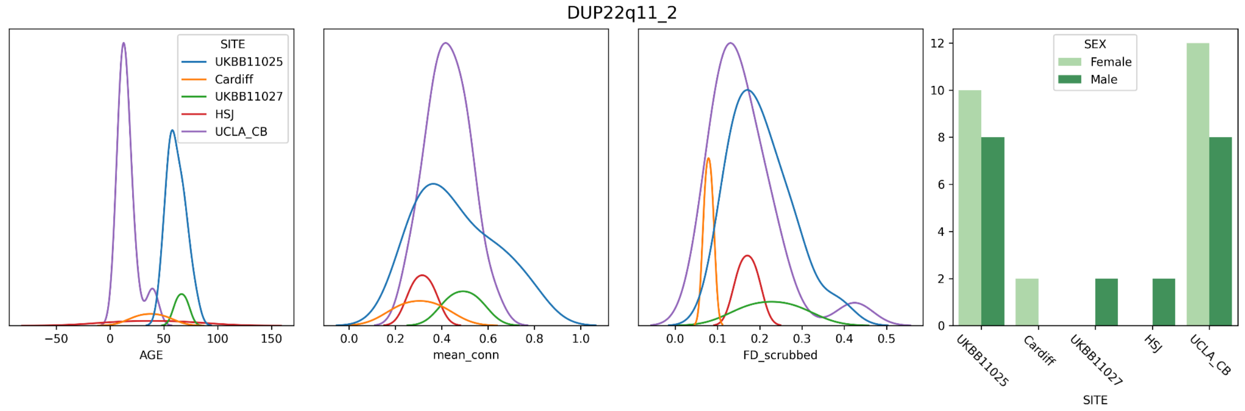

Figure 49 - Distribution of confounding variables (age, global signal (mean_conn), head motion (FD_scrubbed), and sex) by scanning site for the DUP 22q11.2 dataset.

A.8 - Ablation Study

In order to evaluate the impact of each dataset on the prediction performance, we performed an ablation study in which we iteratively dropped a single dataset from the set of tasks and repeated the MTL prediction experiments from sections 3.1.3, 3.1.4 and 3.2. Specifically, we conducted 14 experiments for sex prediction and 18 for age prediction (one for each site of data collection dropped). For automatic diagnosis we conducted 11 experiments (one for each condition dataset dropped). Training was performed as described in the methods (section 2.6). This analysis did not identify any dramatic effect of a single site, however excluding some sites did improve on the MTL accuracy, compared to ST (for example Svip2 for sex prediction), although we did not test the statistical significance of such improvements which would need to be adjusted for the very large number of ablation experiments performed here.

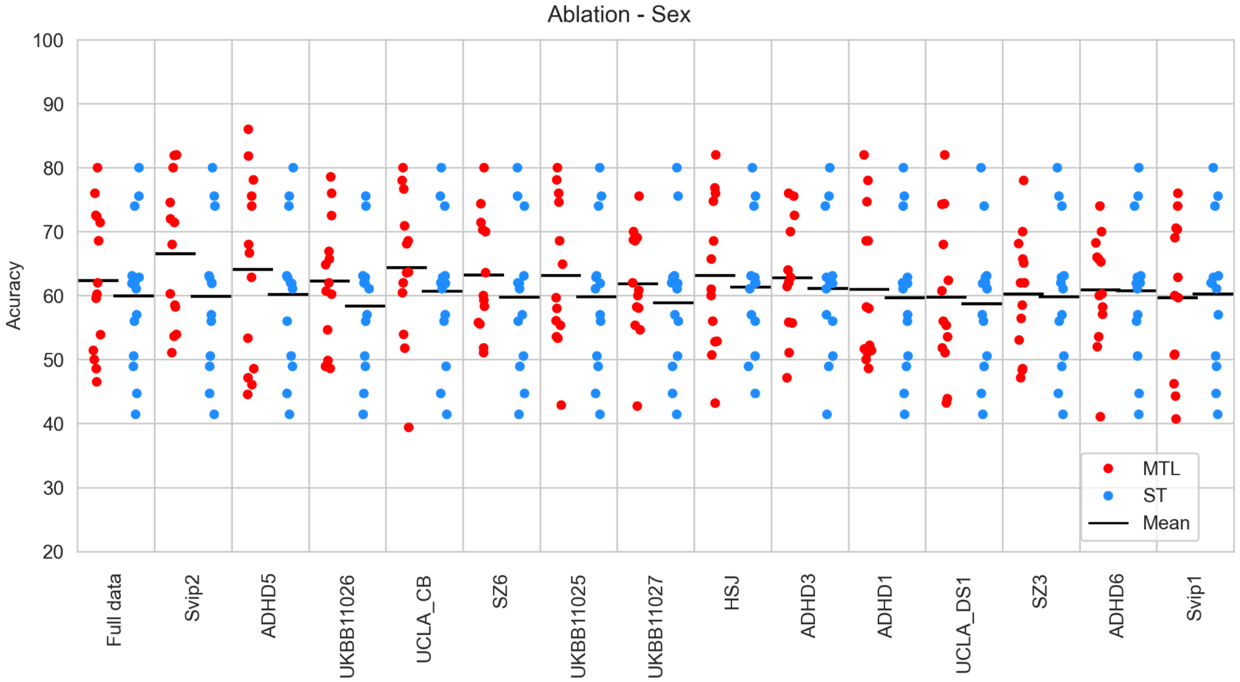

Figure 50 - Distribution of accuracy of sex prediction across tasks using single (ST) vs. multi-task learning (MTL) in a varied collection of sites. The x axis represents the data collection site which is dropped from the set of prediction tasks, except for the first column which shows results using the full dataset. The y axis shows the accuracy of prediction. For each removed dataset, the red points show prediction accuracy distribution for the tasks using the MLPconn architecture in MTL, and the blue points show prediction accuracy distribution on the tasks trained using the MLPconn architecture in ST.

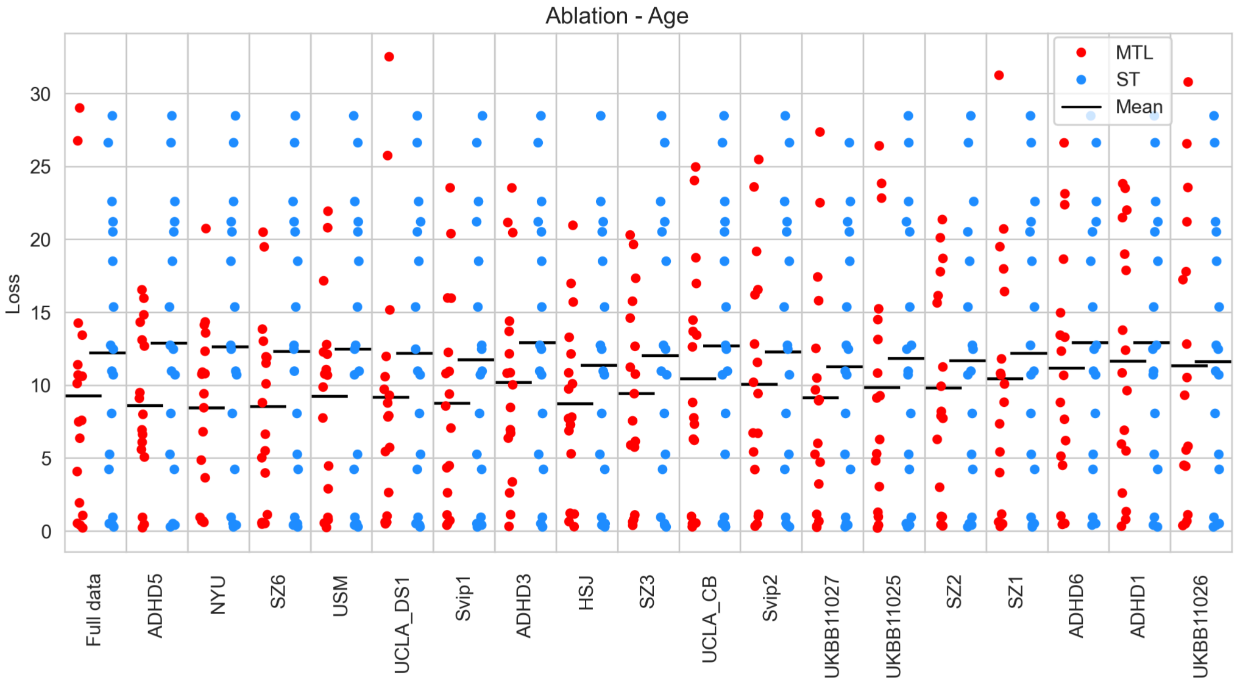

Figure 51 - Distribution of Mean Squared Error (MSE) of age prediction across tasks using single (ST) vs. multi-task learning (MTL) in a varied collection of sites. The x axis represents the data collection site which is dropped from the set of prediction tasks, except for the first column which shows results using the full dataset. The y axis shows the MSE of prediction. For each removed dataset, the red points show prediction error distribution for the tasks using the MLPconn_reg architecture in MTL, and the blue points show prediction error distribution on the tasks trained using the MLPconn_reg architecture in ST.

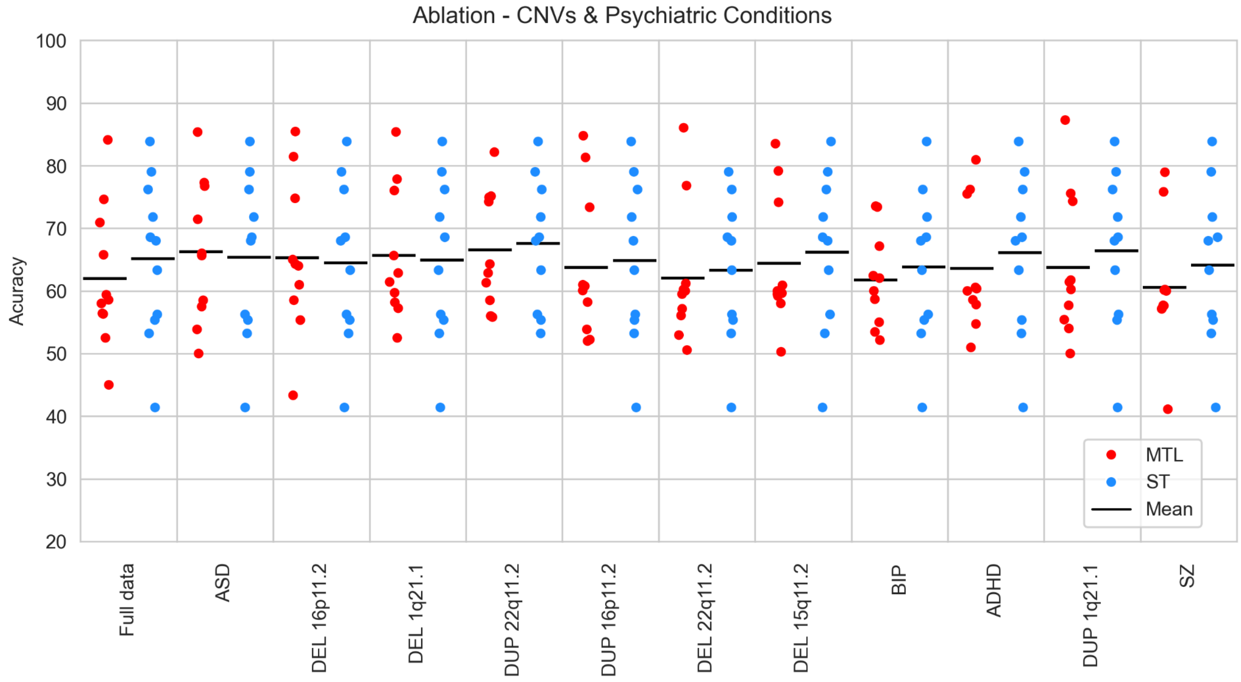

Figure 52 - Distribution of accuracy of automated diagnosis across tasks using single (ST) vs. multi-task learning (MTL) in a varied collection of sites. The x axis represents the condition which is dropped from the set of prediction

tasks, except for the first column which shows results using the full dataset. The y axis shows the accuracy of prediction. For each removed dataset, the red points show prediction accuracy distribution for the tasks using the MLPconn architecture in MTL, and the blue points show prediction accuracy distribution on the tasks trained using the MLPconn architecture in ST.

A.9 - Model Parameter Variations

Here we present the results of a sensitivity analysis in which we varied the parameters of our primary models (MLPconn for classification and MLPconn_reg for regression) in order to evaluate the impact on the performance of MTL in each setting (predicting age, sex and conditions). Training was performed as described in the methods (section 2.6).

The MLPconn_deeper model is a version of the MLPconn model with two additional layers of width 64, one in the shared part of the model and another in the task specific part. The resulting configuration is 2080-256-64-64-64-2. For regression, the output layer is modified to have a single output so that the configuration becomes: 2080-256-64-64-64-1. The input to the model is the connectome vector.

The MLPconn_shorter model is a version of the MLPconn model with the two layers in the shared portion of the model replaced by a single layer with an intermediate width. The resulting configuration is 2080-128-2. For regression, the output layer is modified to have a single output so that the configuration becomes: 2080-128-1. The input to the model is the connectome vector.

The MLPconn_wider model is a version of the MLPconn model with layers that are double the width. The configuration is 2080-512-128-2. For regression, the output layer is modified to have a single output so that the configuration becomes: 2080-512-128-1. The input to the model is the connectome vector.

The MLPconn_thinner model is a version of the MLPconn model with layers that are half the width. The configuration is 2080-128-32-2. For regression, the output layer is modified to have a single output so that the configuration becomes: 2080-128-32-1. The input to the model is the connectome vector.

Regarding sex prediction across cohorts (Figure 53), we observed improved accuracy using MTL over ST, consistently across all variants of architecture. Regarding age prediction across cohorts (Figure 54), we observed improved accuracy (lower error) using MTL over ST for all but one architecture variant: MLPconn_deeper. This suggests that this highly parameterized model may be overfitting in the data regime where it is being trained. Finally, regarding diagnosis across psychiatric conditions and genetic variants (Figure 55), we observed decreased accuracy using MTL over ST for all but one variant: MLPconn_shorter, although the gains in this case are very marginal. This result suggests we may have over-parameterized our primary model MLPconn for this task, but still fails to demonstrate an advantage to MTL on this application. Overall, we found that the conclusions of our study are quite robust to the specific architectural choices we made for MLPconn.

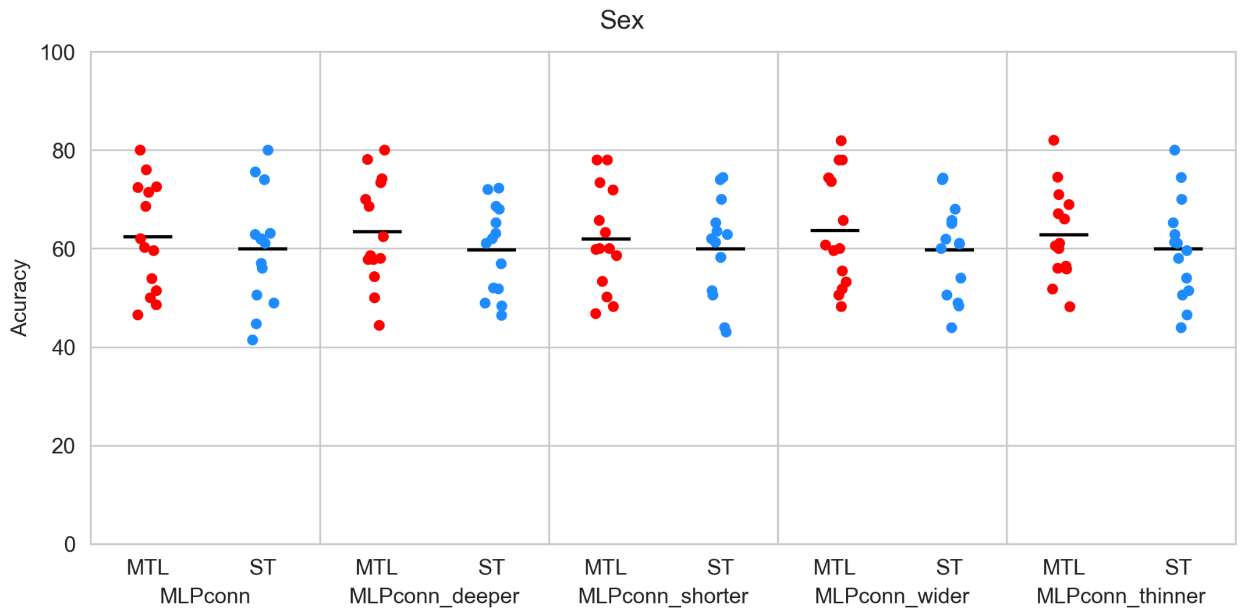

Figure 53 - Distribution of accuracy of sex prediction across model variations using single (ST) vs. multi-task learning (MTL). The x axis represents the model variations. The y axis shows the accuracy of prediction. For each model, the red points show prediction accuracy distribution for the tasks in MTL, and the blue points show prediction accuracy distribution on the tasks trained in ST.

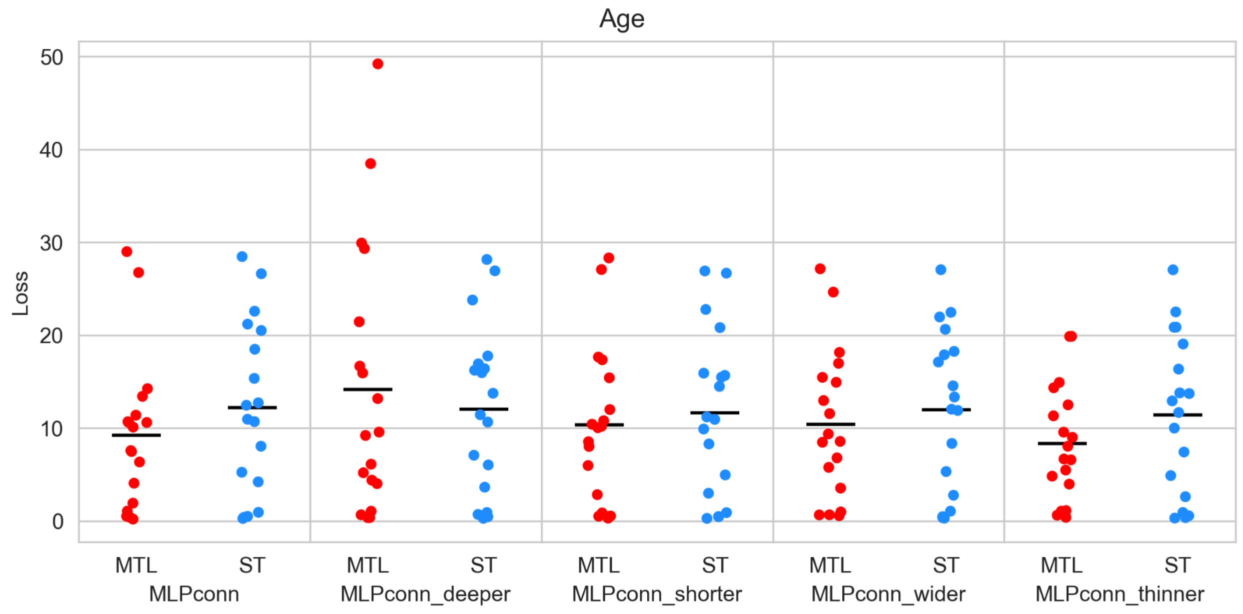

Figure 54 - Distribution of Mean Squared Error (MSE) of age prediction across model variations using single (ST) vs. multi-task learning (MTL). The x axis represents the model variations. The y axis shows the error of prediction. For each model, the red points show prediction accuracy distribution for the tasks in MTL, and the blue points show prediction accuracy distribution on the tasks trained in ST.

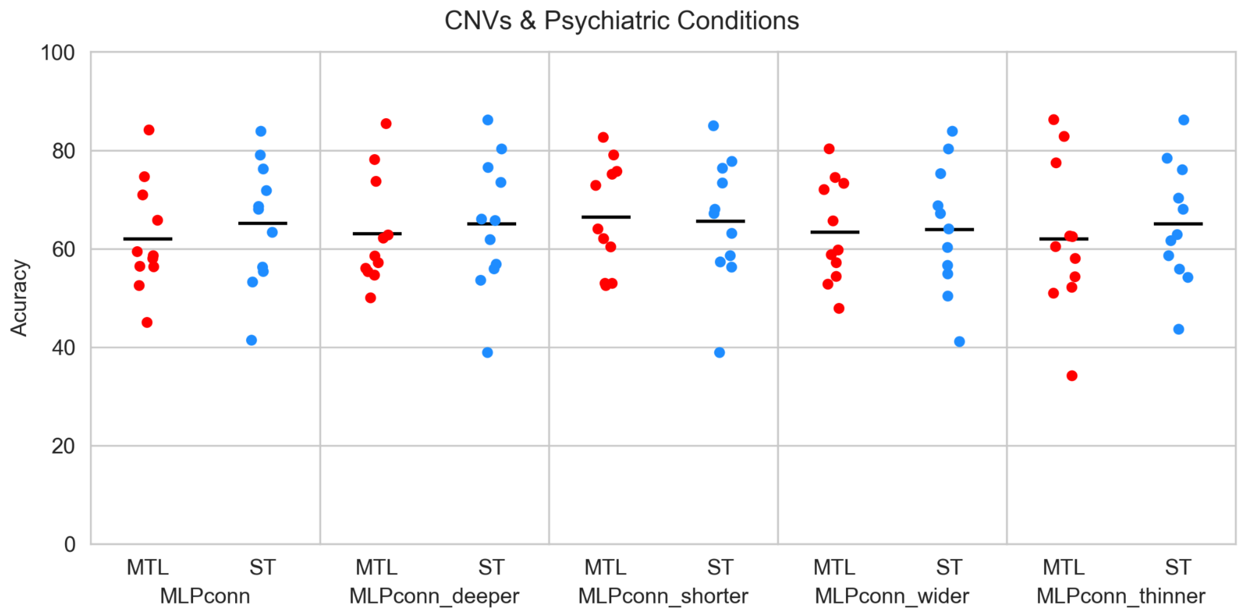

Figure 55 - Distribution of accuracy of automated diagnosis across model variations using single (ST) vs. multi-task learning (MTL). The x axis represents the model variations. The y axis shows the accuracy of prediction. For each model, the red points show prediction accuracy distribution for the tasks in MTL, and the blue points show prediction accuracy distribution on the tasks trained in ST.

A.10 - CNN Variations

Here we present the results of a sensitivity analysis in which we varied the parameters of our convolutional neural network model (CNN) in order to vary the format of the input to the model and evaluate the impact on the performance of MTL for automatic diagnosis. Our primary model uses a random permutation of the connectome as input, and therefore does not consider spatial information. The variations we present here (CNN_64 and CNN_clust, defined below) take as input the full connectome and a reordering of the full connectome determined by a functional clustering respectively. We chose the variants to test the impact of using formats that preserve more information about the spatial layout and functional similarity of the connectome. Training was performed as described in the methods (section 2.6).

The CNN_64 model is a convolutional neural network with a very similar architecture to the main CNN model (see methods section 2.5). Rather than taking the upper triangle of the symmetric connectome matrix (2080 values) randomly permuted and formatted into a 40 x 52 matrix as input, it takes as input the full 64x64 connectome matrix with regions as ordered in the original parcellation, which respects spatial groupings of the regions (Urchs et al. 2017). The model consists of a first convolution layer with 256 filters of shape 8 x 8, followed by two dense layers of 64 hidden units. The output layer has 2 units for binary classification. Batch normalisation (Ioffe and Szegedy 2015) is applied after each layer.

The CNN_clust model has the same architecture as the CNN_64 model, but it takes as input the 64x64 connectome with regions grouped according to a hierarchical clustering performed using ward's criterion (Ward 1963) over the mean connectome taken over all the subjects in our dataset.

We observed that MTL reached lower accuracy than ST for all choices, when applied to diagnosis across psychiatric conditions and genetic variants (Figure 56), consistent with the main results of our paper. We also observed that the model variants working directly on the 64x64 connectomes achieved similar performance to our main CNN architecture for STL, but performed much worse for MTL. We were not able to interpret that result. A possible culprit for the bad performance could be the inability to mix information from multiple networks in the convolutional layers, as neighbouring connections are by construction in similar networks. The fully connected layers also feature a much higher number of parameters in CNN_64 and CNN_clust, which may lead to overfitting as suggested by our experience of architecture variants (Figure 55).

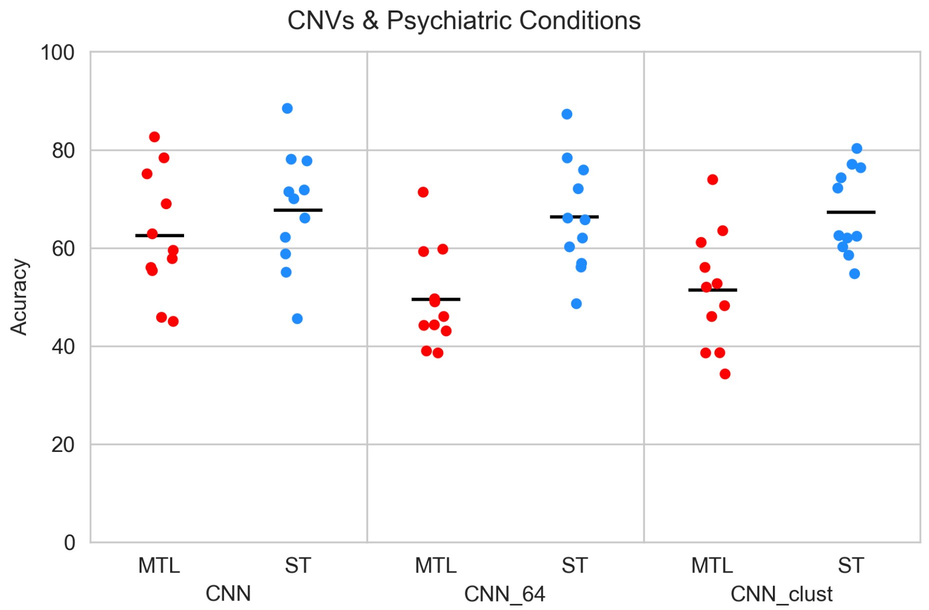

Figure 56 - Distribution of accuracy of automatic diagnosis across model variations using single (ST) vs. multi-task learning (MTL). The x axis represents the model variations. The y axis shows the accuracy of prediction. For each model, the red points show prediction accuracy distribution for the tasks in MTL, and the blue points show prediction accuracy distribution on the tasks trained in ST.

References

Benjamini, Yoav, and Yosef Hochberg. 1995. “Controlling the False Discovery Rate: A Practical and Powerful Approach to Multiple Testing.” *Journal of the Royal Statistical Society* 57 (1): 289–300.

Bzdok, Danilo, and John P. A. Ioannidis. 2019. “Exploration, Inference, and Prediction in Neuroscience and Biomedicine.” *Trends in Neurosciences* 42 (4): 251–62.

Huang, Zhi-An, Rui Liu, and Kay Chen Tan. 2020. “Multi-Task Learning for Efficient Diagnosis of ASD and ADHD Using Resting-State fMRI Data.” In *2020 International Joint Conference on Neural Networks (IJCNN)*, 1–7.

Huang, Zhi-An, Rui Liu, Zexuan Zhu, and Kay Chen Tan. 2022. “Multitask Learning for Joint Diagnosis of Multiple Mental Disorders in Resting-State fMRI.” *IEEE Transactions on Neural Networks and Learning Systems*, 1–15.

Ioffe, Sergey, and Christian Szegedy. 2015. “Batch Normalization: Accelerating Deep Network Training by Reducing Internal Covariate Shift.” *arXiv [cs.LG]*. arXiv. http://arxiv.org/abs/1502.03167.

Li, Jingwei, Ru Kong, Raphaël Liégeois, Csaba Orban, Yanrui Tan, Nanbo Sun, Avram J. Holmes, Mert R. Sabuncu, Tian Ge, and B. T. Thomas Yeo. 2019. “Global Signal Regression Strengthens Association between Resting-State Functional Connectivity and Behavior.” *NeuroImage* 196 (August): 126–41.

Lo, Adeline, Herman Chernoff, Tian Zheng, and Shaw-Hwa Lo. 2015. “Why Significant Variables Aren’t Automatically Good Predictors.” *Proceedings of the National Academy of Sciences* 112 (45): 13892–97.

Ma, Jiaqi, Zhe Zhao, Xinyang Yi, Jilin Chen, Lichan Hong, and Ed H. Chi. 2018. “Modeling Task Relationships in Multi-Task Learning with Multi-Gate Mixture-of-Experts.” In *Proceedings of the 24th ACM SIGKDD International Conference on Knowledge Discovery & Data Mining*, 1930–39. KDD ’18. New York, NY, USA: Association for Computing Machinery.

Masoudnia, Saeed, and Reza Ebrahimpour. 2014. “Mixture of Experts: A Literature Survey.” *Artificial Intelligence Review* 42 (2): 275–93.

Moreau, Clara A., Annabelle Harvey, Kuldeep Kumar, Guillaume Huguet, Sebastian G. W. Urchs, Elise A. Douard, Laura M. Schultz, et al. 2023. “Genetic Heterogeneity Shapes Brain

Connectivity in Psychiatry." *Biological Psychiatry* 93 (1): 45–58.

Moreau, Clara A., Kuldeep Kumar, Annabelle Harvey, Guillaume Huguet, Sebastian Urchs, Laura M. Schultz, Hanad Sharmarke, et al. 2022. "Brain Functional Connectivity Mirrors Genetic Pleiotropy in Psychiatric Conditions." *Brain: A Journal of Neurology*, September. https://doi.org/10.1093/brain/awac315.

Nahm, Francis Sahngun. 2022. "Receiver Operating Characteristic Curve: Overview and Practical Use for Clinicians." *Korean Journal of Anesthesiology* 75 (1): 25–36.

Shmueli, Galit. 2010. "To Explain or to Predict?" *Schweizerische Monatsschrift Fur Zahnheilkunde = Revue Mensuelle Suisse D'odonto-Stomatologie / SSO* 25 (3): 289–310.

Taha, Abdel Aziz, and Allan Hanbury. 2015. "Metrics for Evaluating 3D Medical Image Segmentation: Analysis, Selection, and Tool." *BMC Medical Imaging* 15 (August): 29.

Urchs, Sebastian, Jonathan Armoza, Yassine Benhajali, Jolène St-Aubin, Pierre Orban, and Pierre Bellec. 2017. "MIST: A Multi-Resolution Parcellation of Functional Brain Networks." *MNI Open Research* 1 (December): 3.

Ward, Joe H., Jr. 1963. "Hierarchical Grouping to Optimize an Objective Function." *Journal of the American Statistical Association* 58 (301): 236–44.

Yan, Chao-Gan, R. Cameron Craddock, Xi-Nian Zuo, Yu-Feng Zang, and Michael P. Milham. 2013. "Standardizing the Intrinsic Brain: Towards Robust Measurement of Inter-Individual Variation in 1000 Functional Connectomes." *NeuroImage* 80 (October): 246–62.
